# Field-based high throughput phenotyping rapidly identifies genomic regions controlling yield components in rice

**DOI:** 10.1038/srep42839

**Published:** 2017-02-21

**Authors:** Paul Tanger, Stephen Klassen, Julius P. Mojica, John T. Lovell, Brook T. Moyers, Marietta Baraoidan, Maria Elizabeth B. Naredo, Kenneth L. McNally, Jesse Poland, Daniel R. Bush, Hei Leung, Jan E. Leach, John K. McKay

**Affiliations:** 1 Bioagricultural Sciences and Pest Management, Colorado State University, Fort Collins, CO, USA; 2 International Rice Research Institute (IRRI), Los Baños, Philippines; 3 Department of Biology, Duke University, Durham, NC, USA; 4 Department of Integrative Biology, University of Texas, Austin, Austin, TX, USA; 5 Departments of Plant Pathology and Agronomy, Kansas State University, Manhattan, KS, USA; 6 Department of Biology, Colorado State University, Fort Collins, CO, USA

## Abstract

To ensure food security in the face of population growth, decreasing water and land for agriculture, and increasing climate variability, crop yields must increase faster than the current rates. Increased yields will require implementing novel approaches in genetic discovery and breeding. Here we demonstrate the potential of field-based high throughput phenotyping (HTP) on a large recombinant population of rice to identify genetic variation underlying important traits. We find that detecting quantitative trait loci (QTL) with HTP phenotyping is as accurate and effective as traditional labor-intensive measures of flowering time, height, biomass, grain yield, and harvest index. Genetic mapping in this population, derived from a cross of an modern cultivar (IR64) with a landrace (Aswina), identified four alleles with negative effect on grain yield that are fixed in IR64, demonstrating the potential for HTP of large populations as a strategy for the second green revolution.

The Green Revolution produced elite crop varieties that partition more fixed carbon in harvested tissues and are adapted to high nutrient input production. Together these strategies resulted in a tripling of grain production for rice and other cereals^1^. In the coming 50 years, demand for cereals is projected to increase faster than the rate of yield increase^2^. Over 40% of the land surface of earth is already dedicated to crop and pastureland and further expansion will negatively impact other ecosystem services^3^, so yield increases must come from improvements in crop production and delivery systems^4^. In some systems, agronomic practices to maximize yields have already been optimized^5^. Together, these observations have led to calls for a second Green Revolution that, like the first Green Revolution, implements paradigm-shifting ideas or technologies to increase the rate of yield gain.

A critical component for accelerating the development of new, improved crop varieties is rapid and precise phenotypic assessment of thousands of breeding lines under field conditions. The lack of phenotyping platforms that can efficiently identify individuals exhibiting rare, optimal genotypes from large populations is a major bottleneck for crop improvement. Furthermore, because many agriculturally important traits are influenced by interactions of genotype and environment, field-scale phenotyping is required^6^,^7^,^8^. Despite technological innovations that characterize genomes quickly and cheaply, and computational methods that continue to improve the analysis of such large data sets, the ability to rapidly and accurately measure plant performance in the field remains a limiting factor in plant breeding and genetics. Field-based high-throughput phenotyping (HTP) is an emerging tool with the potential to accelerate genetic discovery and to identify genetic combinations that will allow more rapid selection of high yielding varieties^9^,^10^,^11^. These platforms have been successfully deployed in cotton^12^ and maize^13^, and the rapid nature of data collection has even enabled analyses of temporally dynamic traits^14^. Here we evaluate the capacity of a new field-based HTP platform to meet these goals in rice, a globally important crop. Using a genotyping-by-sequencing approach followed by QTL analysis, we identify several alleles with significant negative effects on grain yield in the semi-dwarf cultivar, IR64, demonstrating the power of combining field-level HTP and large genetic populations for identifying large and moderate effect polymorphisms of relevance to breeding.

## Field-based HTP versus manual phenotyping

**Figure 1:**
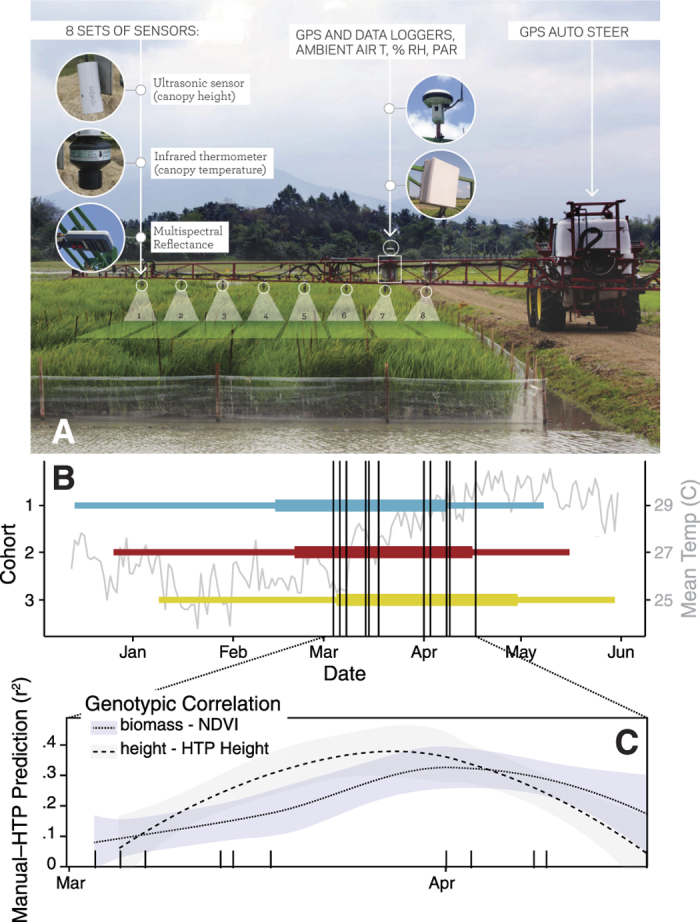
(**A**) Tractor based HTP platform with eight sets of canopy sensors mounted on a 24 m boom. (**B**) Experimental design: each horizontal bar represents a repeated planting cohort from sowing to harvest, with the range of plot heading dates (80% of plants flowered) represented by thicker bars. Vertical lines indicate HTP sampling dates. These are plotted over mean daily air temperature (second y axis) during the growing season. (**C**) Genetic correlations (r^2^) between two pairs of manual and HTP traits over time. Correlations for each HTP sampling date (hash marks on x-axis) and cohort are represented by loess-smoothed lines and shaded 95% confidence intervals. See Fig. S2 for correlations between each HTP and manual traits, for each sampling date for every HTP trait, in every cohort.

For rapid assessment of a suite of important yield component traits under field conditions, we developed and deployed a tractor-based HTP platform that incorporated a precision global navigation satellite system to position and track proximal sensors for rapid, nondestructive plot-level assessment (Fig. 1A). We based our HTP platform on one that was successfully deployed to phenotype cotton^12^,^14^. Using tractor-mounted multispectral reflectance and ultrasonic sensors, we collected canopy height (HTP height), canopy temperature depression (CTD), and several reflectance ratios (NDVI, NDRE, and Chla). Previous studies have shown that these reflectance ratios correlate well with important traits including yield, canopy cover, biomass, leaf area index, flowering time, nitrogen status, plant growth and responses to biotic/abiotic stress^15^,^16^,^17^. Canopy temperature has been used as a proxy for water status in response to drought and canopy stomatal conductance^18^,^19^.

We applied the HTP system to a rice population of 1,516 recombinant inbred lines (RILs) derived from a cross between a high-yielding semi-dwarf cultivar (IR64) and a high biomass landrace (Aswina). The RILs were grown in an incomplete split-plot design in the field at the International Rice Research Institute (IRRI). The population was too large to simultaneously transplant and harvest as a fully replicated design, so experimental blocking occurred across three adjacent fields with sowing and planting dates staggered by two weeks (Fig. 1B). We measured days to heading, plant height, total biomass and grain yield manually following the standard, labor- and time-intensive protocols used at IRRI. In parallel, we collected HTP data at a rate of 3000 plots per hour. Although our experimental plan called for weekly HTP sampling, in practice we were limited by weather and technical issues to 11 time points spanning different growth stages throughout the growing season (Fig. 1B and Table S1). Our QTL analyses proved robust to this sampling scheme, as described below.

Manual phenotyping of a relatively simple trait like height in rice can be done by two experienced professionals at approximately 45 plots per hour, or approximately 200 hours for all plots in this study. Other phenotypes require multiple visits (e.g. phenology) or are significantly more labor-intensive (e.g. biomass or grain yield). On the other hand, the eleven HTP sampling dates took, in total, approximately 18 hours of labor (by the tractor driver, at a rate of 3000 plots/hour). Furthermore, the ease of HTP allows for the tracking of plant responses over time, which is particularly useful for phenotyping responses to biotic and abiotic stress. We also note that each final data point (value for a single RIL in one cohort) is of an order of magnitude higher dimensionality in the HTP data (~15 measured points per plot) versus the manual data (3 plants per plot). Collectively, these gains represent significant savings in time and labor for substantially more data.

Phenotypic and genetic correlations between manual and HTP phenotypes varied across time, with many of the strongest correlations occurring during later flowering stages (Figs 1B,C and S2). Similarly, phenotypic correlations across different sampling dates for each HTP trait varied substantially, primarily decreasing with increasing time between sampling dates (Fig. S2). This was expected since manual data was only collected at flowering (DTH) and at the end of the season (height, biomass, and grain weight), whereas HTP data collection began prior to flowering. Because of this, much of the variation in phenotypic correlations is likely due to temporal dynamics in trait development and expression, and HTP therefore enables the study and genetic dissection of these kinds of traits. For example, some early season HTP correlations with end of season height and biomass were strongly negative but became increasingly positive over time (NDRE, CHLA, in cohorts 2–3), suggesting the canopies of tall, high biomass lines developed more slowly during early vegetative growth (Fig. S2).

## HTP validation and QTL analysis

**Figure 2:**
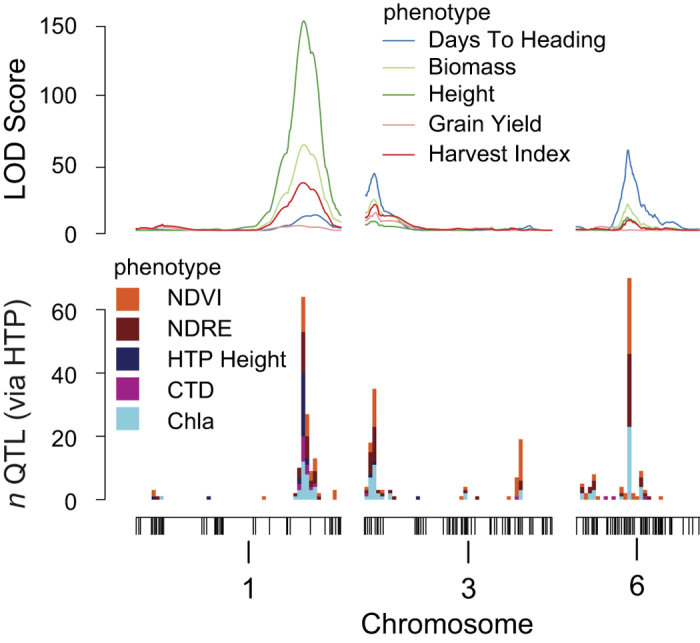
Manually-measured traits are genetically correlated with HTP traits at three major pleiotropic QTL. Top panel plots QTL LOD scores for manually measured traits (LS means across three cohorts for days to heading, biomass, and height, and cohort 3 values for grain and harvest index). Bottom panel is a stacked histogram of statistically significant QTL peak locations for five HTP traits across all 11 measurement days and three cohorts (N = 165 QTL models). See Supplementary Figure S3 and Table S4 for QTL across all chromosomes.

**Figure 3:**
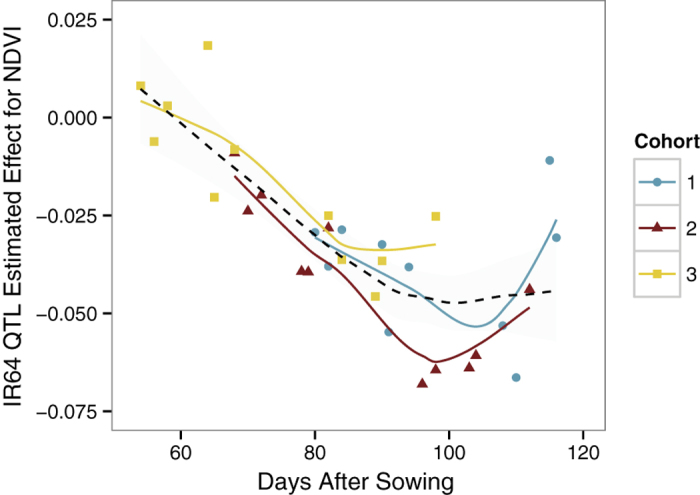
The NDVI effect size of QTL 3@10.0 (Chromosome#@cM) changes consistently over time. The best fit line for each cohort is plotted along with the overall best fit line (dashed black). QTL 3@10.0 has the highest effect on grain yield in this study.

High phenotypic variation, heritability (H^2^ = 0.46–0.83, Table S2), and transgressive segregation was observed for all manually measured traits (Fig. S1). While correlations and H^2^ of HTP traits were more variable over the growth period (Table S3), QTL identified using manually measured phenotypes (generally collected at harvest; >100 days after sowing; DAS) were consistently observed at multiple HTP time points (Figs 2 and S3), and were detected as early as 54 DAS (Fig. 3). The ability to accurately estimate QTL from more than 4500 plots in a 1.5 ha field trial with one pass of the HTP tractor in under two hours opens a new frontier in plant genetics and breeding.

In addition to the greatly enhanced throughput, HTP enabled time-series characterization of QTL over plant development and maturity (Fig. 3). We identified 559 QTL on 12 chromosomes in our population (Table S4), and we highlight some here. Using manually measured grain yield data collected on one cohort, and multispectral (Normalized Difference Vegetation Index; NDVI) data measured on three cohorts, we observed a major QTL on Chr 3 that co-localizes with the largest yield QTL as early as 78 DAS. The effect size increased over time, and then decreased as plants started to senesce. Phenotyping over time provided an estimate of the optimal developmental windows for genetic signal in each phenotype using HTP. We observed 80–100 DAS as the best developmental window in which to identify QTL for NDVI that predict yield (Fig. 3) indicating that HTP can offer rapid, early prediction of phenotypes and ultimately improved selection efficiency. The staggered cohort experimental design allowed us to demonstrate that this window is relatively robust over the three cohorts, despite each experiencing somewhat different developmental environments (Fig. 1B).

A highly significant QTL on Chromosome 1 was identified for plant height (LOD score in all cohorts for manually measured height was 183), (Table S4). This QTL had an additive effect from the Aswina allele of 28 cm ± 0.8 SE and represented 54% of the total variance for plant height in the population. IR64 harbors the semidwarf1 (sd1) allele for the gibberellin (GA) 20-oxidase enzyme (OsGA20ox2, Os01g66100) located on the long arm of chromosome 1 resulting in the semidwarf stature^20^. We speculate here that Aswina does not carry this allele, based on several pieces of evidence. First, Aswina is 61 cm taller than IR64 on average. Second, the bimodal distribution and the high broad-sense heritability of the plant height phenotype support the hypothesis that lines in the RIL population will be short or tall largely dependent on which allele they have at this locus. The QTL reported on chromosome 1 falls between the nearest markers at 169 cM (34,689,333 bp) and 172 cM (39,470,914 bp) and where the SD1 gene on the Nipponbare reference genome would be located (38,382,382–38,385,504 bp). Furthermore, the additive effect of the Aswina allele is +28 cm.

**Figure 4:**
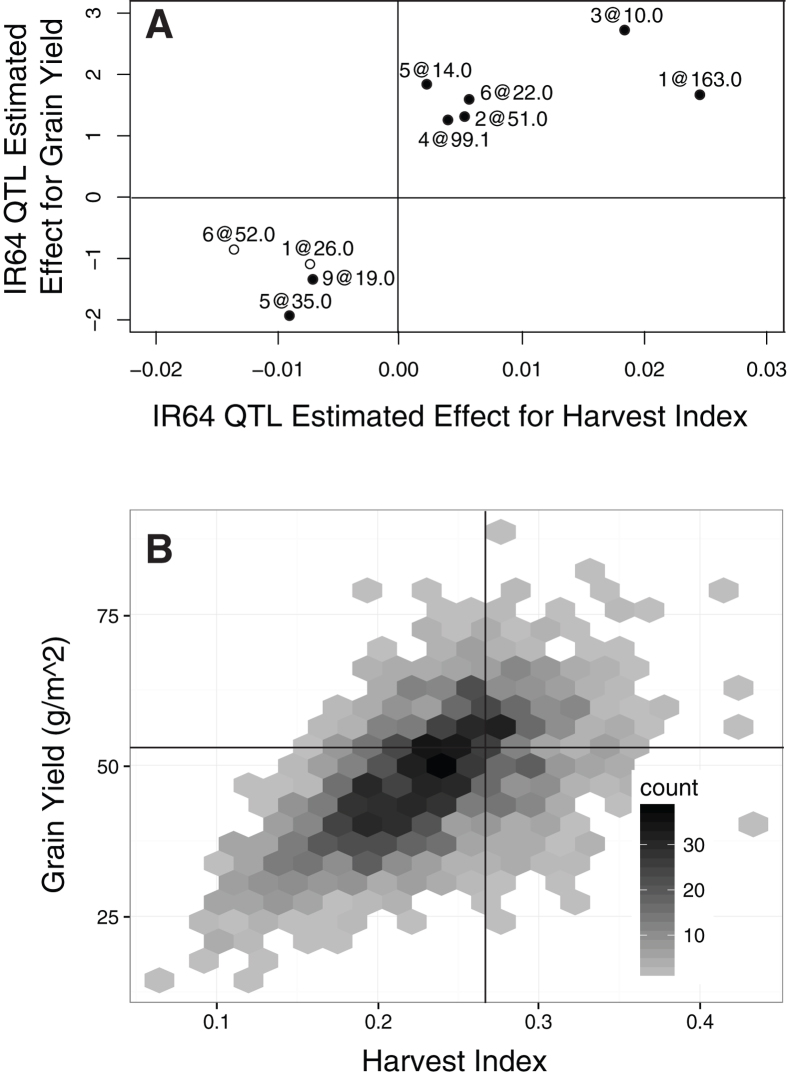
(**A**) The estimated effect of IR64 QTL for grain yield versus for harvest index. Most QTL are pleiotropic and affect both traits (black circles), but two only affect harvest index (open circles). Consensus QTL locations for the two traits are indicated by Chromosome#@cM. (**B**) The joint distribution of breeding values for grain yield and harvest index show the value of recombination in this large population: transgressive segregation for both grain yield and harvest index. The density of RIL LSMean values is represented with grey hexes, and the four quadrants are centered at the trait means of IR64.

We also found four independent QTL where the IR64 (modern cultivar) allele exhibits a negative yield effect (Fig. 4A lower left quadrant), showing the potential to make larger yield gains by using field-based HTP on large breeding populations. Six beneficial QTL contributed to high yield and harvest index (HI) (Fig. 4A top right quadrant). The effect sizes of the beneficial QTL were greater on average, suggesting weak selection and limited recombination in the pedigree leading to IR64. By using HTP, we simultaneously identified variants with effects on a variety of agronomic traits including height, biomass and grain yield. The variation in this large population also demonstrated the power to identify rare high yielding individual lines, for example, with grain and HI highlighted in the top right quadrant (Fig. 4B). The ability to detect and exploit natural adaptive variation in the rice gene pool is potentially transformative to the public sector pipeline to improve the rate of yield gain in rice.

## Conclusions

We demonstrate the utility of field based HTP to identify genomic regions that influence yield and yield components in rice. We report a new, large mapping population of rice with over 1500 recombinant inbred lines, and describe our methods for field based HTP. Our results show that our field based HTP in this large population is able to identify the genomic regions controlling yield and yield components, non-destructively and in a fraction of the time. Our method and other field based HTP will allow efficient screening of large populations and accurate estimations of breeding values and effects of individual loci. HTP can offer rapid, early prediction of phenotypes leading to better selection of lines to enter into the next breeding cycle. Combined with other innovations, field-based HTP provides a technology needed to advance the rate of yield gain to meet the demand of a growing human population.

## Materials and Methods

### Plant materials, genotyping, linkage map construction

Bulked F7 RILs (1,751) were derived from eight F1 plants from a cross (IR 91968) between two *Oryza sativa* indica varieties, IR64 (IRGC 117268) × Aswina (IRGC 117281), by single seed descent. We performed genotyping-by-sequencing using a restriction digest approach (*PstI* and *MspI*)^21^. Sequence was processed in TASSEL 4.3.5, build date January 16, 2014^22^ (except for the MergeIdenticalTaxaPlugin step, where TASSEL 3.0.165 build date January 1, 2014 was used) by alignment to the Nipponbare reference sequence version 7^23^ using bowtie2 version 2.1.0^24^. Putative SNPs were filtered for 1) less than 40% missing data, 2) for duplicate SNPs pairs with 99% or greater similarity, one SNP of each pair was discarded. Next, individuals with greater than 15% heterozygous calls were filtered out. Based on the reduced number of individuals, SNPs with calls in less than 80% of the population were discarded. SNPs with segregation distortion were then filtered out (<30% or >70%) resulting in 4046 SNPs and 986 individuals that were used in subsequent analyses. A genetic map was constructed using an iterative process that involved creating genetic maps in Joinmap, and filtering them in R Statistical Computing 3.1.2^25^. In the first iteration, a genetic map was constructed in Joinmap 4 (www.kyazma.nl). Groups were chosen using the recombination frequency approach with a start, end, and step of 0.250, 0.050, and −0.050 respectively. Maps were created using the maximum likelihood approach with the system defaults. This map was then exported and filtered. One of each pair of individuals that were 99% similar was then discarded to improve processing time. The remaining analyses were conducted in R/qtl 1.30–4 or 1.36–6^26^. This was followed by discarding individuals with abnormally high numbers of total crossovers (countXO function). Next, the LOD score of double crossovers was calculated and these marker groups were discarded (top.errorlod function). Markers that significantly changed the length of the linkage groups were discarded (droponemarker function). This filtered dataset was used to create a revised genetic map in Joinmap using the same parameters as before. The revised map was exported and markers with high segregation distortion were discarded (pickMarkerSubset function), and marker order was refined with a window of 5 cM (ripple function). For each marker, we calculated composite score with equal weights for the amount of missing data and the degree of segregation distortion. Within each 1 cM window the marker with the highest composite score was retained. Strong linkage disequilibrium was observed among a set of markers on Chr 12 and several other chromosomes. Retaining such linked markers reduces the power and accuracy of QTL mapping. Therefore, we opted to discard these markers. This dataset was again used in Joinmap to produce a genetic map with the same parameters as before, except the step was changed to 0.300, followed by another round of filtering in R (pickMarkerSubset, ripple functions). The final map contained 433 SNPs and 943 RILs.

### Experimental design

All 1,751 Recombinant Inbred Lines were grown at the Experiment Station at the International Rice Research Institute (IRRI), Los Baños, Laguna, Philippines (lat 14°10′11.69″N, long 121°14′38.63″E, 21 m elevation) in 2012 Wet Season (WS). Lines that were visually segregating within a plot were removed and seed was bulked at F7 for each of the remaining 1,526 lines. Approximately 200 seeds from each RIL were sown in a rectangular plastic container in the greenhouse. At 18–21 days after sowing seedlings were pulled out from soil, bundled and brought to the field for transplanting. Seeds were germinated December 2012 and transplanted January 2013.

The experiment utilized two adjacent 2500 m^2^ fields separated by a 3 m wide bund for traversing with the tractor. A total of 1568 entries included the following: 1516 F7 derived RILs (1474 RILs had single plots while 42 RILs were planted in two plots), the two parents Aswina & IR64, and other standard IRRI check lines. Each plot consisted of 5 × 6 (30) hills, on 20 cm spacing (1.2 m^2^ plots). Entries were grouped into 8 blocks based on height in order to minimize shading by adjacent plots since plant height was known to vary by as much as one meter. Each block included one entry that was replicated to estimate within block effects. No common entry was included in all blocks. Together, the 8 blocks comprised 49 rows of 16 plots wide in two adjacent fields. To estimate repeatability and observe variation over the growing season, this experiment was repeated in two additional plantings (hereafter referred to as “cohorts”) in adjacent 2500 m^2^ fields, with planting dates offset by about two weeks between each cohort. Molluscicide was applied immediately after transplanting. Fertilizer was hand broadcast at 160-30-30 with split application of N which was 30 kg N per hectare from complete fertilizer^14^ and 30 kg N per hectare from UREA (46-0-0) applied right after transplanting then 40 kg N and 60 kg N per hectare applied at 20 and 50 days after transplanting from UREA (46-0-0).

### Manual phenotyping

Days to heading (DTH) was recorded when 80% of the plants in the plot had flowered. Three plants from the middle of each plot were randomly sampled for plant height using a meter stick, final fresh vegetative biomass (excluding panicle) using lab scales, and panicle mass at maturity. Panicle mass was only collected on the 3^rd^ cohort due to time and labor constraints. Plot data from plots with less than 13 plants per plot were discarded and plot means were calculated for each plot. Harvest index was calculated as the ratio of panicle mass to total aboveground biomass including panicle mass.

### High throughput phenotyping

High throughput phenotyping (HTP) was conducted using a tractor based system similar to^12^ but using a 24 m boom and 8 sets of canopy level sensors on one side of the boom (Fig. 1A). The platform utilized a high precision real time kinematic GPS (GS15, Leica Geosystems; Heerbrugg, Germany) for automated tractor guidance and georeferencing of sensor data. Each set of canopy sensors included a 3-band reflectance sensor (Crop Circle ACS470, Holland Scientific; Lincoln, NE USA), an infrared canopy temperature sensor (SI-131, Apogee Instruments; Logan, UT, USA), and an ultrasonic canopy height sensor (dB3, Pulsar; Malvern, UK). Measured spectral reflectance bands included near infrared (NIR, 760–800 nm), red edge (RE, 730 nm) and red (R, 670 nm). The platform included a sensor for monitoring air temperature and humidity (HMP110, Vaisala; Vantaa, Finland), and photosynthetically active radiation (SQ-110, Apogee Instruments; Logan, UT USA). Sensor data was recorded using two data loggers (CR5000, Campbell Scientific; Logan, UT USA and GeoScout GLS-420, Holland Scientific; Lincoln, NE USA). The sensors were aligned to traverse along the center of each plot, simultaneously recording data for 8 plots at a time at a rate of 5 samples per second (5 Hz). The sensors were maintained at a constant height above the ground, at about 0.50 m above the canopy. The tractor was operated at a speed of 1.0 kph, resulting in an overall throughput of about 3000 plots per hour. The platform was operated 1–2 times per week (11 times) from March 4, 2013 to April 17, 2013. Intermittent rain and mechanical failures resulted in variation in intervals between HTP measures.

Daily raw HTP data was post processed using R 3.2.2 as follows: plot level data was extracted based on GPS location, excluding data within 0.20 m of the borders of each plot (i.e., excluding the border plants of each plot). Daily reflectance data was normalized to the first sensor based on the mean values per run across a field on that day. The following three vegetation indices were calculated using the three normalized reflectance bands: NDVI = (NIR − R)/(NIR + R); NDRE = (NIR − RE)/(NIR + RE); and Chla = (NIR/RE) − 1^16^,^17^.

Canopy temperature sensors were normalized to the first sensor based on the mean water temperature of unplanted plots at the ends of each field across all sampling dates. Canopy height was calculated as the difference between the height of the sensor above ground as determined by GPS, and the sensor measured distance to the canopy. Canopy temperature depression was calculated as the difference in air temperature and canopy temperature.

The data were filtered in the following ways to improve consistency and to remove anomalous data: All excluded if tractor speed <0.3 kph or moving in reverse; All excluded if the number of plants per plot <13; Sensor excluded if number of samples per plot <4; Sensor excluded if coefficient of variance (CV) per plot >0.5; Canopy temperature excluded if T < 22.0 C or T > 40.0 C; Canopy height excluded if Ht < 0.10 m or Ht > 2.60 m. Postprocessed data was output as daily CSV files for statistical analysis.

### QTL mapping

QTL mapping was performed with R/qtl 1.30–4 or 1.36–6^26^ a package for R Statistical Computing. In R/qtl, single QTL scans were completed with Haley-Knott (HK)^27^ algorithms. Genotype probabilities were estimated at 1 cM intervals with the Kosambi mapping function. Significance LOD thresholds were derived from 1,000 permutations according to a genome-wide type I error rate of 5%. The possibility of interacting QTL was examined with the scantwo function in R/qtl and relevant penalties for interaction were employed. Stepwise model selection was performed with a maximum of 10 QTL and penalties of 0.1%. QTL interval was defined as 1.5 LOD from the peak LOD score.

R code is currently hosted at bitbucket.org (https://bitbucket.org/paultanger/rilpopr) and code as well as original data files will be deposited into http://dx.doi.org/10.5061/dryad.53bj8 and www.datadryad.org for publication and availability.

## Additional Information

**How to cite this article**: Tanger, P. *et al*. Field-based high throughput phenotyping rapidly identifies genomic regions controlling yield components in rice. *Sci. Rep.* **7**, 42839; doi: 10.1038/srep42839 (2017).

**Publisher's note:** Springer Nature remains neutral with regard to jurisdictional claims in published maps and institutional affiliations.

## Supplementary Material

Supplementary Information
