# Supplementary material for: Field-based high throughput phenotyping rapidly identifies genomic regions controlling yield components in rice
Source: Sci Rep. 2017 Feb 21;7:42839. doi: 10.1038/srep42839 (PMC5318881; doi:10.1038/srep42839)
Supplement: Supplementary Information [file srep42839-s1.pdf]

Title: Field-based high throughput phenotyping rapidly identifies genomic regions controlling yield components in rice

Authors: Paul Tanger [1]†, Stephen Klassen [2]†, Julius P. Mojica [1,3], John T. Lovell [4], Brook T. Moyers [1], Marietta Baraoidan [2], Maria Elizabeth B. Naredo [2], Kenneth L. McNally [2], Jesse Poland [5] Daniel R. Bush [6], Hei Leung [2], Jan E. Leach [1]*, John K. McKay [1]*

Supplementary Table 1. Summary of HTP data collection showing the Days After Sowing for each cohort on the 11 final HTP sampling dates. Two sets of cross-cohort comparable DAS are highlighted.

	Days After Sowing (DAS)		
Date	Cohort 1	Cohort 2	Cohort 3
3/4/13	80	68	54
3/6/13	82	70	56
3/8/13	84	72	58
3/14/13	90	78	64
3/15/13	91	79	65
3/18/13	94	82	68
4/1/13	108	96	82
4/3/13	110	98	84
4/8/13	115	103	89
4/9/13	116	104	90
4/17/13	124	112	98

Supplementary Table 2. Heritability components of manually measured traits. Grain yield and HI are not shown because only one cohort of data was collected. SD is standard deviation.

Phenotype	Component	Variance	SD	H^2
Days to heading	Line	53.48	7.31	59%
	Cohort	10.38	3.22	11%
	Residual	27.14	5.21	30%
Biomass	Line	1119.17	33.45	46%
	Cohort	332.97	18.25	14%
	Residual	998.53	31.60	41%
Height	Line	910.76	30.18	83%
	Cohort	77.32	8.79	7%
	Residual	109.26	10.45	10%

Supplementary Table 3. HTP Phenotype heritability by date and cohort.

	Cohort	3/4/13	3/6/13	3/8/13	3/14/13	3/15/13	3/18/13	4/1/13	4/3/13	4/8/13	4/9/13	4/17/13
**NDVI**	**1**	54.5	44.6	48.1	58.2	46.8	57.7	71.3	77.1	44.2	67.3	no data
**NDRE**	**1**	41.2	27.2	21.0	53.7	57.2	53.7	47.5	53.0	6.0	50.4	no data
**Chla**	**1**	46.1	31.9	29.4	63.6	58.7	54.7	46.9	51.5	6.0	51.9	no data
**HT**	**1**	no data	8.1	no data	38.9	53.9	31.8	42.6	63.2	67.5	80.9	no data
**CTD**	**1**	no data	56.7	no data	58.4	83.7	55.3	40.0	52.9	33.1	86.6	no data
**NDVI**	**2**	41.7	29.4	1.3	10.7	16.3	26.4	36.6	70.4	57.4	45.4	34.4
**NDRE**	**2**	59.1	56.0	29.0	21.4	22.9	15.4	18.3	26.6	30.0	17.6	36.1
**Chla**	**2**	52.1	56.5	39.5	24.1	23.4	20.1	18.8	28.3	30.5	16.6	35.0
**HT**	**2**	no data	0.0	no data	8.5	12.5	37.3	52.2	49.2	65.2	0.0	0.0
**CTD**	**2**	no data	43.6	no data	63.6	80.7	7.8	46.7	72.9	70.3	80.1	74.6
**NDVI**	**3**	15.1	24.9	26.4	39.8	30.4	46.6	27.9	35.8	51.9	53.7	58.7
**NDRE**	**3**	18.3	30.3	23.8	35.0	64.9	46.0	58.4	37.4	7.0	40.8	41.2
**Chla**	**3**	18.7	26.8	23.3	36.6	66.9	48.3	59.9	40.4	7.9	41.7	40.4
**HT**	**3**	no data	0.0	no data	41.2	27.3	26.9	39.9	82.6	75.8	65.1	6.7
**CTD**	**3**	no data	60.2	no data	77.7	77.6	34.3	0.0	26.6	28.1	0.0	64.2

Supplementary Table 4. All Significant QTL identified in the study. Note that the QTLs listed here are the result of model selection and differ than those displayed in Figure 2 and S3 which display scan1 QTL. For the HTP phenotypes, the cohort and DAS are identified. For manually measured phenotypes, the QTL are the LSmeans of all 3 cohorts.

phenotype	cohort	chromosome	position	LOD	% variance	effect	DAS
Chla	1	1	165.0	9.42	4.72	0.034	80
Chla	1	2	125.0	3.51	1.73	-0.017	80
Chla	1	6	52.4	5.50	2.73	0.022	80
Chla	1	1	169.0	15.36	7.45	0.038	82
Chla	1	2	125.0	3.17	1.49	-0.018	82
Chla	1	3	1.0	4.09	1.92	-0.021	82
Chla	1	6	52.4	7.76	3.69	0.025	82
Chla	1	3	5.2	4.70	2.35	-0.023	84
Chla	1	4	133.8	3.29	1.64	-0.021	84
Chla	1	6	52.4	7.45	3.75	0.030	84
Chla	1	1	182.0	9.26	4.15	-0.023	90
Chla	1	3	5.2	8.32	3.72	-0.024	90
Chla	1	4	100.2	3.52	1.55	-0.015	90
Chla	1	6	52.4	23.10	10.75	0.036	90
Chla	1	1	182.0	24.25	10.97	-0.050	91
Chla	1	3	5.2	10.40	4.52	-0.029	91
Chla	1	4	99.1	4.95	2.12	-0.019	91

Chla	1	6	53.0	28.82	13.22	0.045	91
Chla	1	1	182.0	14.05	5.22	-0.032	94
Chla	1	3	6.0	21.45	8.13	-0.039	94
Chla	1	3	103.0	2.87	1.04	-0.013	94
Chla	1	4	105.8	3.47	1.25	-0.015	94
Chla	1	6	7.0	4.40	1.59	0.017	94
Chla	1	6	52.4	38.54	15.30	0.050	94
Chla	1	11	22.3	3.30	1.19	0.014	94
Chla	1	1	177.4	3.89	1.36	-0.015	108
Chla	1	3	9.0	65.96	27.38	-0.061	108
Chla	1	3	157.0	2.87	1.00	-0.013	108
Chla	1	6	52.4	20.59	7.54	0.034	108
Chla	1	11	21.0	3.21	1.12	0.014	108
Chla	1	1	174.0	9.92	3.42	-0.025	110
Chla	1	3	8.0	69.76	28.37	-0.059	110
Chla	1	3	157.0	2.84	0.96	-0.013	110
Chla	1	6	52.0	20.64	7.32	0.032	110
Chla	1	11	22.0	2.83	0.96	0.012	110
Chla	1	3	9.0	30.51	14.87	-0.027	115
Chla	1	6	53.0	8.29	3.80	0.014	115
Chla	1	11	22.0	3.70	1.67	0.010	115
Chla	1	1	179.0	3.01	1.23	-0.009	116
Chla	1	3	9.0	42.51	19.37	-0.027	116
Chla	1	6	52.4	12.55	5.27	0.012	116
Chla	1	11	22.3	5.73	2.36	0.012	116
Chla	1	11	64.0	2.75	1.13	-0.010	116
Chla	2	1	168.0	10.19	5.12	0.053	68
Chla	2	3	26.0	3.49	1.73	0.032	68
Chla	2	12	64.7	2.86	1.41	0.022	68
Chla	2	1	175.0	9.47	4.39	0.294	70
Chla	2	1	178.0	7.85	3.62	-0.226	70
Chla	2	3	27.0	3.37	1.53	0.029	70
Chla	2	12	59.0	4.34	1.98	0.025	70
Chla	2	1	168.0	16.82	7.80	0.064	72
Chla	2	2	11.0	5.66	2.55	0.022	72
Chla	2	3	18.8	2.13	0.95	0.018	72
Chla	2	6	45.9	6.18	2.79	0.020	72
Chla	2	12	64.7	3.88	1.74	0.024	72
Chla	2	1	162.0	6.93	4.08	0.023	78
Chla	2	2	2.0	7.98	4.72	0.025	78
Chla	2	6	52.4	5.32	3.12	0.025	78
Chla	2	1	173.0	7.52	4.07	0.012	79
Chla	2	2	3.0	4.59	2.47	0.022	79
Chla	2	6	52.4	13.20	7.27	0.031	79
Chla	2	1	169.0	11.37	6.63	0.025	82
Chla	2	6	52.4	13.83	8.13	0.033	82
Chla	2	1	172.0	8.85	3.91	0.014	96
Chla	2	2	122.6	2.96	1.29	-0.020	96
Chla	2	3	6.0	15.20	6.83	-0.047	96
Chla	2	6	53.0	19.66	8.93	0.048	96
Chla	2	1	180.0	12.90	4.97	-0.026	98
Chla	2	2	120.3	2.91	1.09	-0.014	98
Chla	2	3	8.0	24.69	9.81	-0.045	98
Chla	2	3	101.0	3.16	1.19	-0.015	98

Chla	2	5	33.0	3.28	1.23	0.015	98
Chla	2	6	52.4	35.59	14.57	0.049	98
Chla	2	1	172.0	17.90	6.84	-0.041	103
Chla	2	3	8.0	27.19	10.65	-0.046	103
Chla	2	4	100.2	5.36	1.98	0.000	103
Chla	2	6	7.0	7.00	2.59	-0.009	103
Chla	2	6	52.4	28.51	11.20	0.044	103
Chla	2	1	174.0	15.73	6.01	-0.039	104
Chla	2	3	8.0	36.37	14.69	-0.058	104
Chla	2	6	15.0	2.97	1.10	-0.016	104
Chla	2	6	52.4	28.34	11.20	0.047	104
Chla	2	1	168.0	9.39	3.51	-0.032	112
Chla	2	3	9.0	50.97	21.34	-0.059	112
Chla	2	6	19.0	7.54	2.80	-0.011	112
Chla	2	6	52.4	19.48	7.48	0.038	112
Chla	2	11	21.0	4.44	1.64	0.018	112
Chla	3	1	167.0	12.32	5.88	0.052	54
Chla	3	2	20.0	4.52	2.11	0.025	54
Chla	3	3	15.0	4.32	2.02	0.025	54
Chla	3	8	72.0	3.03	1.41	-0.024	54
Chla	3	1	168.0	14.45	6.90	0.063	56
Chla	3	2	20.3	3.77	1.75	0.025	56
Chla	3	3	15.0	3.09	1.43	0.024	56
Chla	3	8	71.0	3.34	1.55	-0.028	56
Chla	3	1	168.0	8.60	4.23	0.056	58
Chla	3	2	18.1	4.95	2.41	0.026	58
Chla	3	2	151.0	4.73	2.30	0.029	58
Chla	3	6	65.5	3.62	1.75	0.030	58
Chla	3	8	74.0	3.70	1.79	-0.033	58
Chla	3	1	170.0	30.78	19.08	0.097	64
Chla	3	6	64.0	3.87	2.18	0.029	64
Chla	3	1	174.0	8.32	4.16	0.639	65
Chla	3	1	175.0	7.54	3.77	-0.527	65
Chla	3	6	67.0	3.67	1.81	0.028	65
Chla	3	1	169.0	47.74	23.23	0.109	68
Chla	3	6	67.0	7.37	3.19	0.030	68
Chla	3	1	169.0	68.72	28.82	0.150	82
Chla	3	2	123.0	3.92	1.38	-0.031	82
Chla	3	3	4.0	3.68	1.29	-0.030	82
Chla	3	6	52.4	13.95	5.04	0.049	82
Chla	3	1	168.0	56.61	25.45	0.109	84
Chla	3	2	122.6	3.52	1.36	-0.024	84
Chla	3	3	4.0	6.29	2.44	-0.033	84
Chla	3	6	52.4	16.87	6.76	0.045	84
Chla	3	1	167.0	15.93	6.95	0.028	89
Chla	3	3	9.0	16.01	6.98	-0.035	89
Chla	3	6	18.2	3.92	1.65	-0.016	89
Chla	3	6	52.4	26.22	11.77	0.041	89
Chla	3	12	28.0	3.07	1.29	0.014	89
Chla	3	1	168.0	17.32	7.48	0.036	90
Chla	3	2	126.5	4.68	1.96	-0.021	90
Chla	3	3	9.0	12.35	5.26	-0.037	90
Chla	3	6	15.7	4.01	1.67	-0.020	90
Chla	3	6	52.4	16.27	7.01	0.037	90

Chla	3	1	24.0	6.64	2.28	0.014	98
Chla	3	3	8.0	44.29	16.80	-0.058	98
Chla	3	3	156.0	3.51	1.20	-0.015	98
Chla	3	6	19.0	13.35	4.67	-0.022	98
Chla	3	6	52.4	22.53	8.07	0.040	98
Chla	3	11	22.3	8.46	2.92	0.018	98
Chla	3	12	29.0	3.90	1.33	0.015	98
CTD	1	1	172.0	11.54	6.02	-0.187	82
CTD	1	1	172.0	4.03	2.46	-0.133	90
CTD	1	1	171.0	22.53	15.50	-0.566	91
CTD	1	1	170.0	33.74	19.50	-0.527	94
CTD	1	11	105.5	2.86	1.49	0.119	94
CTD	1	1	177.4	3.31	3.19	-0.225	108
CTD	1	9	68.0	3.18	3.06	0.226	108
CTD	1	1	180.0	7.25	6.72	-0.289	110
CTD	1	5	104.0	2.89	2.62	0.168	110
CTD	1	1	167.0	13.93	10.94	-0.403	115
CTD	1	6	69.8	3.14	2.35	0.155	115
CTD	1	8	94.0	3.29	2.95	-0.168	116
CTD	2	1	166.0	4.57	5.61	0.260	70
CTD	2	3	153.0	3.23	2.32	-0.209	78
CTD	2	5	104.0	5.57	4.04	-0.273	78
CTD	2	1	169.0	3.68	2.32	-0.151	79
CTD	2	5	36.0	2.94	1.85	0.111	79
CTD	2	5	102.0	5.02	3.18	-0.150	79
CTD	2	5	108.0	4.50	5.03	-0.345	96
CTD	2	3	0.0	3.13	2.80	-0.196	104
CTD	2	5	104.4	6.18	3.65	-0.209	112
CTD	2	6	32.0	3.36	1.97	-0.158	112
CTD	3	1	169.0	28.59	15.10	0.419	56
CTD	3	12	26.7	3.51	1.72	0.114	56
CTD	3	1	168.0	42.91	36.71	0.954	64
CTD	3	1	168.0	12.71	8.38	0.428	65
CTD	3	1	171.0	13.54	7.98	0.494	68
CTD	3	1	172.0	5.21	5.78	-0.274	89
CTD	3	1	171.0	39.60	19.45	0.516	98
CTD	3	6	39.0	2.93	1.30	0.116	98
HTPheight	1	1	170.0	14.12	16.89	-11.280	82
HTPheight	1	3	52.4	3.13	3.47	-4.064	82
HTPheight	1	1	169.0	30.33	18.95	-14.960	90
HTPheight	1	1	169.0	43.15	28.59	-18.143	91
HTPheight	1	1	170.0	57.12	30.36	-18.116	94
HTPheight	1	1	169.0	15.93	14.81	-12.426	108
HTPheight	1	3	6.0	3.32	2.89	-4.544	108
HTPheight	1	1	171.0	11.24	13.24	-11.183	110
HTPheight	1	3	10.8	3.04	3.38	-4.610	110
HTPheight	1	1	167.0	10.99	13.73	-11.922	115
HTPheight	1	5	5.0	2.77	3.26	-4.793	115
HTPheight	1	1	171.0	15.12	18.23	-12.782	116
HTPheight	2	1	72.0	2.96	8.57	4.039	70
HTPheight	2	1	174.0	12.26	8.92	-7.273	78
HTPheight	2	2	5.5	2.96	2.07	3.084	78
HTPheight	2	5	7.0	5.01	3.54	-2.630	78
HTPheight	2	8	108.7	6.52	4.63	-3.510	78

HTPheight	2	1	168.0	33.71	24.13	-12.413	79
HTPheight	2	1	169.0	21.90	16.98	-11.105	82
HTPheight	2	1	169.0	28.31	24.88	-14.857	96
HTPheight	2	4	126.8	6.51	5.06	-1.663	96
HTPheight	2	12	55.9	7.36	5.74	2.451	96
HTPheight	2	1	169.0	19.62	21.82	-14.472	98
HTPheight	2	1	170.0	10.79	12.29	-11.326	103
HTPheight	2	1	16.0	3.81	3.58	5.359	104
HTPheight	2	1	168.0	17.39	17.68	-14.714	104
HTPheight	2	1	166.0	6.16	5.46	-7.887	112
HTPheight	3	1	169.0	3.95	6.32	-4.704	64
HTPheight	3	1	170.0	13.74	11.88	-7.230	65
HTPheight	3	1	168.0	17.62	12.73	-8.602	68
HTPheight	3	1	170.0	24.73	23.96	-14.578	82
HTPheight	3	5	45.0	2.80	2.38	4.386	82
HTPheight	3	1	172.0	23.92	29.67	-18.180	84
HTPheight	3	1	170.0	18.50	23.19	-17.290	89
HTPheight	3	1	168.0	24.47	24.08	-16.226	90
HTPheight	3	1	171.0	4.99	3.91	-6.103	98
NDRE	1	1	165.0	8.46	4.26	0.007	80
NDRE	1	2	125.0	3.24	1.61	-0.003	80
NDRE	1	6	52.4	5.68	2.84	0.005	80
NDRE	1	1	169.0	14.27	6.94	0.008	82
NDRE	1	2	125.0	3.03	1.43	-0.004	82
NDRE	1	3	1.0	4.19	1.98	-0.005	82
NDRE	1	6	52.4	7.82	3.73	0.005	82
NDRE	1	1	186.0	5.10	2.44	-0.006	84
NDRE	1	3	5.2	3.80	1.81	-0.005	84
NDRE	1	4	99.1	3.88	1.85	-0.005	84
NDRE	1	6	53.0	10.00	4.85	0.007	84
NDRE	1	8	81.8	3.19	1.52	-0.004	84
NDRE	1	1	182.0	10.59	4.72	-0.007	90
NDRE	1	3	5.2	8.03	3.55	-0.006	90
NDRE	1	4	103.8	3.55	1.55	-0.004	90
NDRE	1	6	52.4	24.12	11.14	0.010	90
NDRE	1	1	182.0	27.84	12.27	-0.014	91
NDRE	1	3	5.2	9.77	4.08	-0.007	91
NDRE	1	4	99.1	5.23	2.16	-0.005	91
NDRE	1	6	53.0	31.67	14.12	0.013	91
NDRE	1	12	7.6	2.69	1.10	0.004	91
NDRE	1	1	182.0	16.08	5.95	-0.009	94
NDRE	1	3	6.0	20.00	7.48	-0.010	94
NDRE	1	3	103.0	2.98	1.06	-0.004	94
NDRE	1	4	105.8	3.56	1.27	-0.004	94
NDRE	1	6	7.0	4.40	1.58	0.005	94
NDRE	1	6	52.4	40.72	16.12	0.014	94
NDRE	1	11	22.3	3.26	1.16	0.004	94
NDRE	1	1	177.4	3.64	1.30	-0.004	108
NDRE	1	3	9.0	62.60	26.22	-0.019	108
NDRE	1	3	157.0	2.90	1.03	-0.004	108
NDRE	1	6	52.4	19.69	7.32	0.011	108
NDRE	1	11	21.0	3.06	1.09	0.004	108
NDRE	1	1	174.0	10.16	3.59	-0.008	110
NDRE	1	3	8.0	66.02	27.26	-0.019	110

NDRE	1	3	157.0	3.15	1.09	-0.004	110
NDRE	1	6	52.0	19.92	7.24	0.010	110
NDRE	1	3	9.0	29.22	14.20	-0.009	115
NDRE	1	6	53.0	8.09	3.71	0.005	115
NDRE	1	11	22.0	3.60	1.63	0.004	115
NDRE	1	1	175.0	2.83	1.16	-0.003	116
NDRE	1	3	9.0	42.68	19.46	-0.010	116
NDRE	1	6	52.4	12.50	5.25	0.004	116
NDRE	1	11	22.3	5.74	2.37	0.004	116
NDRE	1	11	64.0	2.77	1.13	-0.003	116
NDRE	2	1	168.0	8.82	4.46	0.012	68
NDRE	2	3	26.0	3.50	1.74	0.008	68
NDRE	2	12	64.7	2.67	1.33	0.005	68
NDRE	2	1	168.0	14.91	7.29	0.013	70
NDRE	2	3	29.0	3.23	1.53	0.007	70
NDRE	2	12	64.7	4.05	1.92	0.005	70
NDRE	2	1	168.0	13.12	6.20	0.013	72
NDRE	2	2	10.0	6.73	3.13	0.006	72
NDRE	2	6	45.9	7.06	3.28	0.005	72
NDRE	2	12	64.7	3.49	1.61	0.005	72
NDRE	2	1	162.0	5.76	3.40	0.004	78
NDRE	2	2	2.0	7.94	4.71	0.006	78
NDRE	2	6	52.0	5.70	3.36	0.006	78
NDRE	2	1	173.0	6.88	3.71	0.002	79
NDRE	2	2	3.0	4.85	2.60	0.005	79
NDRE	2	6	52.4	13.94	7.68	0.007	79
NDRE	2	1	169.0	9.87	5.76	0.005	82
NDRE	2	6	52.4	14.22	8.41	0.007	82
NDRE	2	1	173.0	7.46	3.31	0.002	96
NDRE	2	2	122.6	2.88	1.26	-0.005	96
NDRE	2	3	6.0	13.83	6.24	-0.011	96
NDRE	2	6	53.0	20.50	9.42	0.012	96
NDRE	2	1	180.0	14.15	5.49	-0.007	98
NDRE	2	3	8.0	25.76	10.31	-0.011	98
NDRE	2	3	114.0	4.89	1.85	-0.003	98
NDRE	2	5	34.0	4.11	1.55	0.004	98
NDRE	2	6	52.4	36.50	15.05	0.013	98
NDRE	2	1	172.0	18.66	7.11	-0.012	103
NDRE	2	3	8.0	26.43	10.29	-0.013	103
NDRE	2	4	100.2	5.44	2.00	0.000	103
NDRE	2	6	7.0	7.15	2.64	-0.003	103
NDRE	2	6	52.4	29.83	11.72	0.013	103
NDRE	2	1	174.0	17.05	6.54	-0.012	104
NDRE	2	3	8.0	34.29	13.76	-0.016	104
NDRE	2	6	14.0	2.89	1.07	-0.005	104
NDRE	2	6	52.4	29.86	11.84	0.014	104
NDRE	2	1	168.0	9.31	3.47	-0.011	112
NDRE	2	3	9.0	50.74	21.20	-0.021	112
NDRE	2	6	19.0	7.48	2.78	-0.004	112
NDRE	2	6	52.4	18.74	7.17	0.013	112
NDRE	2	11	21.0	4.23	1.56	0.006	112
NDRE	3	1	167.0	11.06	5.38	0.014	54
NDRE	3	2	20.0	3.92	1.87	0.006	54
NDRE	3	3	16.0	4.58	2.19	0.007	54

NDRE	3	1	168.0	10.95	5.39	0.015	56
NDRE	3	2	20.3	3.18	1.54	0.006	56
NDRE	3	3	15.0	2.75	1.32	0.006	56
NDRE	3	1	168.0	5.88	3.04	0.013	58
NDRE	3	6	66.0	3.06	1.57	0.008	58
NDRE	3	8	72.0	3.35	1.72	-0.009	58
NDRE	3	1	171.0	28.65	17.88	0.022	64
NDRE	3	6	64.0	3.88	2.21	0.007	64
NDRE	3	1	173.0	7.85	4.06	0.050	65
NDRE	3	1	176.0	6.02	3.09	-0.028	65
NDRE	3	6	67.0	3.86	1.97	0.006	65
NDRE	3	1	169.0	40.50	20.13	0.022	68
NDRE	3	6	69.0	7.19	3.25	0.006	68
NDRE	3	1	169.0	68.59	28.75	0.029	82
NDRE	3	2	123.0	4.30	1.51	-0.006	82
NDRE	3	3	4.0	4.02	1.42	-0.006	82
NDRE	3	6	52.4	12.70	4.57	0.009	82
NDRE	3	1	168.0	54.95	24.79	0.023	84
NDRE	3	2	122.6	3.58	1.39	-0.005	84
NDRE	3	3	4.0	6.57	2.58	-0.007	84
NDRE	3	6	52.4	15.76	6.34	0.009	84
NDRE	3	1	167.0	14.53	6.25	0.007	89
NDRE	3	3	9.0	16.46	7.13	-0.009	89
NDRE	3	6	18.2	3.89	1.63	-0.004	89
NDRE	3	6	52.4	17.63	7.66	0.009	89
NDRE	3	6	73.0	2.73	1.14	0.004	89
NDRE	3	12	28.0	3.23	1.35	0.004	89
NDRE	3	1	168.0	15.62	6.75	0.008	90
NDRE	3	2	126.5	4.61	1.93	-0.005	90
NDRE	3	3	9.0	12.55	5.38	-0.009	90
NDRE	3	6	15.7	4.28	1.79	-0.005	90
NDRE	3	6	52.4	16.06	6.95	0.009	90
NDRE	3	1	23.0	5.84	2.02	0.004	98
NDRE	3	3	8.0	43.74	16.69	-0.018	98
NDRE	3	3	156.0	3.73	1.28	-0.005	98
NDRE	3	6	16.0	13.01	4.58	-0.007	98
NDRE	3	6	52.4	21.04	7.57	0.012	98
NDRE	3	11	22.3	8.10	2.82	0.006	98
NDRE	3	12	29.0	4.25	1.46	0.005	98
NDVI	1	1	179.0	42.02	18.08	-0.030	80
NDVI	1	2	9.0	5.54	2.16	0.010	80
NDVI	1	3	144.9	5.51	2.14	0.008	80
NDVI	1	3	156.0	4.17	1.62	-0.011	80
NDVI	1	6	55.0	10.32	4.07	0.011	80
NDVI	1	7	126.3	7.63	2.99	-0.011	80
NDVI	1	8	40.0	3.55	1.37	0.008	80
NDVI	1	8	71.0	5.87	2.29	-0.012	80
NDVI	1	1	173.0	57.24	23.64	-0.037	82
NDVI	1	2	9.0	4.43	1.58	0.008	82
NDVI	1	3	6.0	3.67	1.31	-0.008	82
NDVI	1	3	156.0	4.39	1.57	-0.009	82
NDVI	1	6	56.0	10.52	3.82	0.014	82
NDVI	1	8	44.0	3.13	1.11	0.008	82
NDVI	1	8	73.0	6.34	2.28	-0.013	82

NDVI	1	1	180.0	32.90	13.51	-0.028	84
NDVI	1	2	10.0	9.06	3.49	0.010	84
NDVI	1	3	5.2	3.54	1.34	-0.008	84
NDVI	1	3	156.0	3.66	1.39	-0.008	84
NDVI	1	6	55.3	14.50	5.66	0.015	84
NDVI	1	8	43.0	4.87	1.86	0.011	84
NDVI	1	8	72.0	13.01	5.06	-0.020	84
NDVI	1	10	85.0	5.07	1.93	0.007	84
NDVI	1	1	177.4	36.21	15.62	-0.029	90
NDVI	1	2	15.6	5.20	2.06	0.011	90
NDVI	1	3	156.4	4.27	1.69	-0.010	90
NDVI	1	6	53.0	9.31	3.73	0.016	90
NDVI	1	7	126.3	7.53	3.01	-0.008	90
NDVI	1	8	39.6	4.48	1.77	0.011	90
NDVI	1	8	75.0	4.20	1.66	-0.012	90
NDVI	1	1	173.0	68.36	30.32	-0.059	91
NDVI	1	3	155.4	4.19	1.53	-0.011	91
NDVI	1	6	7.0	3.30	1.20	0.010	91
NDVI	1	6	53.0	19.53	7.47	0.024	91
NDVI	1	1	179.0	42.19	16.95	-0.039	94
NDVI	1	2	15.6	3.62	1.31	0.010	94
NDVI	1	3	156.0	5.91	2.15	-0.013	94
NDVI	1	6	19.0	6.16	2.25	0.014	94
NDVI	1	6	52.4	26.95	10.39	0.028	94
NDVI	1	1	171.0	43.94	13.63	-0.058	108
NDVI	1	3	9.0	78.99	27.05	-0.062	108
NDVI	1	3	155.4	4.66	1.30	-0.015	108
NDVI	1	5	31.2	2.88	0.80	0.011	108
NDVI	1	6	53.0	28.98	8.63	0.040	108
NDVI	1	1	170.0	55.81	18.31	-0.069	110
NDVI	1	3	8.0	64.25	21.59	-0.058	110
NDVI	1	3	155.4	3.61	1.03	-0.014	110
NDVI	1	4	95.8	2.78	0.79	-0.012	110
NDVI	1	6	53.0	23.35	7.00	0.036	110
NDVI	1	3	9.0	36.41	18.73	-0.025	115
NDVI	1	6	52.4	14.32	6.88	0.017	115
NDVI	1	11	22.0	3.39	1.58	0.009	115
NDVI	1	1	171.0	22.91	8.01	-0.030	116
NDVI	1	3	8.0	58.02	22.37	-0.034	116
NDVI	1	6	52.4	15.38	5.27	0.018	116
NDVI	1	11	22.3	5.57	1.86	0.012	116
NDVI	1	11	69.0	2.79	0.92	-0.011	116
NDVI	1	12	54.0	9.04	3.04	0.014	116
NDVI	2	1	203.3	3.15	1.55	-0.013	68
NDVI	2	2	11.0	6.20	3.07	0.016	68
NDVI	2	3	156.0	6.44	3.20	-0.019	68
NDVI	2	6	48.0	5.62	2.78	0.013	68
NDVI	2	1	184.0	13.11	5.87	-0.026	70
NDVI	2	2	11.0	6.54	2.88	0.016	70
NDVI	2	2	129.0	3.98	1.74	0.013	70
NDVI	2	3	156.0	5.19	2.27	-0.014	70
NDVI	2	5	41.0	2.94	1.28	0.011	70
NDVI	2	5	102.0	3.43	1.50	-0.012	70
NDVI	2	6	63.0	3.64	1.59	0.013	70

NDVI	2	8	85.0	4.97	2.18	-0.014	70
NDVI	2	1	182.0	10.77	4.62	-0.021	72
NDVI	2	2	11.0	13.63	5.90	0.020	72
NDVI	2	2	131.0	4.61	1.95	0.013	72
NDVI	2	3	156.0	6.56	2.79	-0.015	72
NDVI	2	6	47.0	10.08	4.32	0.014	72
NDVI	2	6	86.8	3.06	1.29	0.010	72
NDVI	2	8	93.0	2.90	1.22	-0.009	72
NDVI	2	1	170.0	33.25	15.86	-0.044	78
NDVI	2	2	7.0	7.76	3.40	0.017	78
NDVI	2	2	129.0	6.55	2.86	0.016	78
NDVI	2	3	10.8	4.72	2.05	-0.013	78
NDVI	2	3	158.0	6.96	3.05	-0.013	78
NDVI	2	5	37.0	5.86	2.56	0.015	78
NDVI	2	5	99.5	3.02	1.30	-0.010	78
NDVI	2	6	52.4	4.76	2.07	0.013	78
NDVI	2	8	95.0	6.82	2.98	-0.014	78
NDVI	2	1	171.0	45.37	20.90	-0.046	79
NDVI	2	2	19.0	5.20	2.12	0.012	79
NDVI	2	2	126.5	6.52	2.67	0.013	79
NDVI	2	3	1.1	3.70	1.50	-0.010	79
NDVI	2	3	156.4	3.68	1.49	-0.010	79
NDVI	2	5	33.0	3.06	1.24	0.009	79
NDVI	2	6	52.0	13.78	5.76	0.016	79
NDVI	2	1	171.0	23.37	11.55	-0.030	82
NDVI	2	2	1.6	4.47	2.08	0.010	82
NDVI	2	2	130.0	6.22	2.91	0.014	82
NDVI	2	3	10.8	3.98	1.85	-0.010	82
NDVI	2	3	156.0	4.76	2.22	-0.011	82
NDVI	2	5	45.0	4.76	2.22	0.012	82
NDVI	2	6	52.0	10.06	4.76	0.017	82
NDVI	2	8	86.2	4.05	1.88	-0.010	82
NDVI	2	1	128.0	5.77	1.61	-0.001	96
NDVI	2	1	172.0	85.34	29.57	-0.073	96
NDVI	2	2	3.1	3.57	0.99	0.010	96
NDVI	2	2	76.0	8.29	2.32	0.009	96
NDVI	2	3	9.0	11.17	3.16	-0.020	96
NDVI	2	3	99.0	3.02	0.84	-0.010	96
NDVI	2	3	157.0	3.14	0.87	-0.010	96
NDVI	2	5	29.0	7.49	2.09	0.016	96
NDVI	2	6	52.0	22.94	6.69	0.028	96
NDVI	2	1	172.0	85.50	30.08	-0.068	98
NDVI	2	2	4.4	4.64	1.31	0.012	98
NDVI	2	3	9.0	11.66	3.34	-0.020	98
NDVI	2	3	103.4	5.21	1.47	-0.003	98
NDVI	2	3	154.0	8.29	2.36	-0.012	98
NDVI	2	5	34.0	6.90	1.95	0.014	98
NDVI	2	6	52.0	30.89	9.33	0.032	98
NDVI	2	7	31.0	3.07	0.86	0.011	98
NDVI	2	1	170.0	59.75	21.68	-0.066	103
NDVI	2	3	8.0	24.35	8.01	-0.034	103
NDVI	2	3	155.4	3.23	1.00	-0.012	103
NDVI	2	4	27.8	7.56	2.38	-0.002	103
NDVI	2	5	57.7	3.25	1.01	0.012	103

NDVI	2	6	52.4	19.40	6.30	0.030	103
NDVI	2	12	52.0	9.92	3.14	0.017	103
NDVI	2	1	170.0	58.16	20.69	-0.064	104
NDVI	2	3	9.0	31.55	10.45	-0.039	104
NDVI	2	3	155.0	5.05	1.56	-0.015	104
NDVI	2	5	28.0	3.59	1.10	0.013	104
NDVI	2	6	52.4	25.76	8.40	0.034	104
NDVI	2	1	168.0	22.60	8.90	-0.045	112
NDVI	2	3	9.0	35.51	14.52	-0.048	112
NDVI	2	5	33.0	3.20	1.19	0.013	112
NDVI	2	6	23.0	5.65	2.12	-0.008	112
NDVI	2	6	53.0	17.65	6.85	0.034	112
NDVI	2	11	21.0	3.28	1.22	0.014	112
NDVI	2	12	49.0	4.12	1.54	0.016	112
NDVI	3	2	20.0	4.57	2.22	0.017	54
NDVI	3	2	149.0	3.03	1.46	0.014	54
NDVI	3	6	66.6	5.13	2.49	0.018	54
NDVI	3	8	101.6	4.76	2.31	-0.017	54
NDVI	3	2	20.3	5.02	2.42	0.017	56
NDVI	3	2	124.5	4.51	2.18	0.016	56
NDVI	3	6	66.0	5.37	2.60	0.018	56
NDVI	3	8	96.0	4.30	2.07	-0.016	56
NDVI	3	2	21.2	3.76	1.85	0.017	58
NDVI	3	2	131.0	4.77	2.34	0.021	58
NDVI	3	6	53.0	7.77	3.85	0.025	58
NDVI	3	8	93.0	4.88	2.40	-0.019	58
NDVI	3	1	170.0	4.45	2.77	0.017	64
NDVI	3	2	125.0	5.03	3.13	0.015	64
NDVI	3	6	54.0	7.45	4.68	0.018	64
NDVI	3	8	79.1	3.35	2.07	-0.012	64
NDVI	3	1	180.0	5.45	3.05	-0.019	65
NDVI	3	2	124.5	5.49	3.08	0.017	65
NDVI	3	6	51.0	9.34	5.30	0.023	65
NDVI	3	2	124.5	3.06	1.58	0.012	68
NDVI	3	6	52.4	8.75	4.59	0.022	68
NDVI	3	8	76.0	5.21	2.71	-0.018	68
NDVI	3	1	180.0	26.44	11.82	-0.023	82
NDVI	3	2	86.0	3.29	1.38	0.008	82
NDVI	3	6	11.0	3.38	1.42	0.008	82
NDVI	3	6	53.0	10.29	4.41	0.015	82
NDVI	3	8	80.2	5.62	2.38	-0.007	82
NDVI	3	1	174.0	34.00	15.89	-0.034	84
NDVI	3	2	151.0	3.98	1.71	0.011	84
NDVI	3	6	10.0	3.34	1.43	0.010	84
NDVI	3	6	52.0	8.68	3.78	0.016	84
NDVI	3	8	80.2	5.69	2.45	-0.009	84
NDVI	3	1	18.5	3.23	1.19	0.009	89
NDVI	3	1	172.0	37.96	15.43	-0.042	89
NDVI	3	1	200.0	6.44	2.39	-0.008	89
NDVI	3	2	68.0	5.03	1.86	0.014	89
NDVI	3	3	16.0	5.68	2.11	-0.013	89
NDVI	3	5	31.0	3.12	1.15	0.009	89
NDVI	3	6	53.0	15.08	5.74	0.020	89
NDVI	3	1	172.0	32.99	12.53	-0.034	90

NDVI	3	1	201.0	6.37	2.25	-0.006	90
NDVI	3	3	10.0	6.83	2.42	-0.013	90
NDVI	3	3	156.0	3.47	1.22	-0.009	90
NDVI	3	5	31.0	8.02	2.85	0.011	90
NDVI	3	6	52.4	9.33	3.33	0.017	90
NDVI	3	6	68.2	6.33	2.24	0.008	90
NDVI	3	1	17.0	6.15	2.10	0.010	98
NDVI	3	1	171.0	10.76	3.71	-0.024	98
NDVI	3	3	7.0	35.43	13.04	-0.038	98
NDVI	3	4	106.7	3.93	1.33	-0.012	98
NDVI	3	6	17.0	12.43	4.31	-0.012	98
NDVI	3	6	47.0	5.11	1.74	0.019	98
NDVI	3	6	58.0	8.90	3.06	0.019	98
NDVI	3	11	22.3	8.11	2.78	0.014	98
NDVI	3	12	49.0	5.15	1.75	0.014	98
DTH	final	1	182.0	31.79	6.65	-2.468	
DTH	final	3	9.0	95.93	23.81	-3.848	
DTH	final	3	158.0	6.19	1.21	-0.948	
DTH	final	5	29.0	5.09	0.99	0.871	
DTH	final	6	18.0	14.01	2.80	-0.890	
DTH	final	6	53.0	97.82	24.41	4.726	
DTH	final	6	79.9	2.92	0.57	0.665	
DTH	final	11	19.0	3.53	0.69	0.699	
DTH	final	12	1.0	6.71	1.32	0.953	
biomass	final	1	171.0	86.15	26.99	-25.601	
biomass	final	3	9.0	40.83	11.33	-14.582	
biomass	final	3	156.4	3.74	0.94	-4.105	
biomass	final	4	107.8	2.97	0.75	-3.615	
biomass	final	5	31.0	6.80	1.73	5.366	
biomass	final	6	52.4	32.76	8.90	12.580	
height	final	1	171.0	183.17	53.62	-28.613	
height	final	2	126.5	5.02	0.90	3.072	
height	final	3	10.0	21.68	4.07	-6.820	
height	final	3	153.0	4.39	0.79	-3.058	
height	final	5	31.2	5.87	1.06	3.308	
height	final	5	104.4	2.87	0.51	-2.349	
height	final	6	52.4	23.82	4.49	7.095	
grain yield	3	1	164.0	4.70	1.93	1.931	
grain yield	3	2	51.0	8.89	3.70	1.271	
grain yield	3	3	10.0	15.27	6.46	3.020	
grain yield	3	4	85.8	5.76	2.37	1.805	
grain yield	3	5	15.0	3.38	1.38	1.486	
grain yield	3	5	35.0	4.95	2.04	-1.775	
grain yield	3	6	20.0	3.17	1.30	1.349	
grain yield	3	8	27.0	5.67	2.34	-0.059	
grain yield	3	9	21.0	4.28	1.76	-1.531	
HI	3	1	26.0	3.79	1.18	-0.011	
HI	3	1	170.0	41.95	14.42	0.046	
HI	3	2	50.3	3.44	1.07	0.010	
HI	3	3	10.0	30.09	10.03	0.031	
HI	3	4	109.1	4.64	1.45	0.012	
HI	3	5	35.0	6.32	1.98	-0.014	
HI	3	6	25.0	4.42	1.38	0.014	
HI	3	6	52.4	14.53	4.65	-0.022	

HI	3	9	16.0	3.56	1.11	-0.011
HI	3	12	8.7	5.16	1.61	-0.009

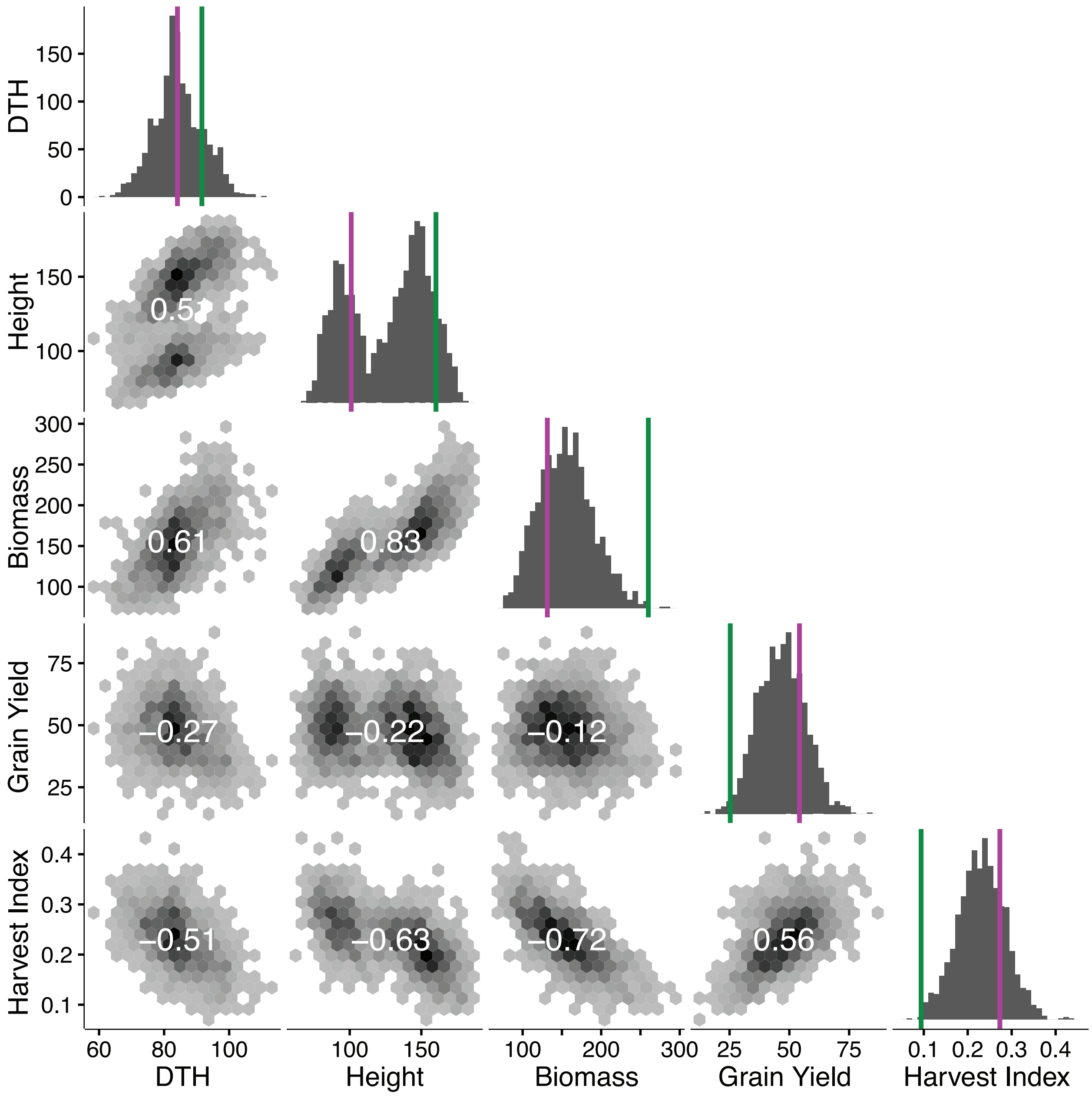

Supplementary Fig. 1. Individual and pairwise joint-density distributions (binned into hexagons) for five manually collected traits (DTH, days to heading; biomass; height; grain yield; HI, harvest index) in cohort 3. Spearman's rank correlation coefficients are reported for the joint-density distributions (all significant at $p < 0.05$). Parental means are plotted for Aswina (green) and IR64 (purple) for each single-trait histogram. Note that the y-axes are for the joint-density distributions and are not the indices for the single-trait histograms.

Figure S2 A.

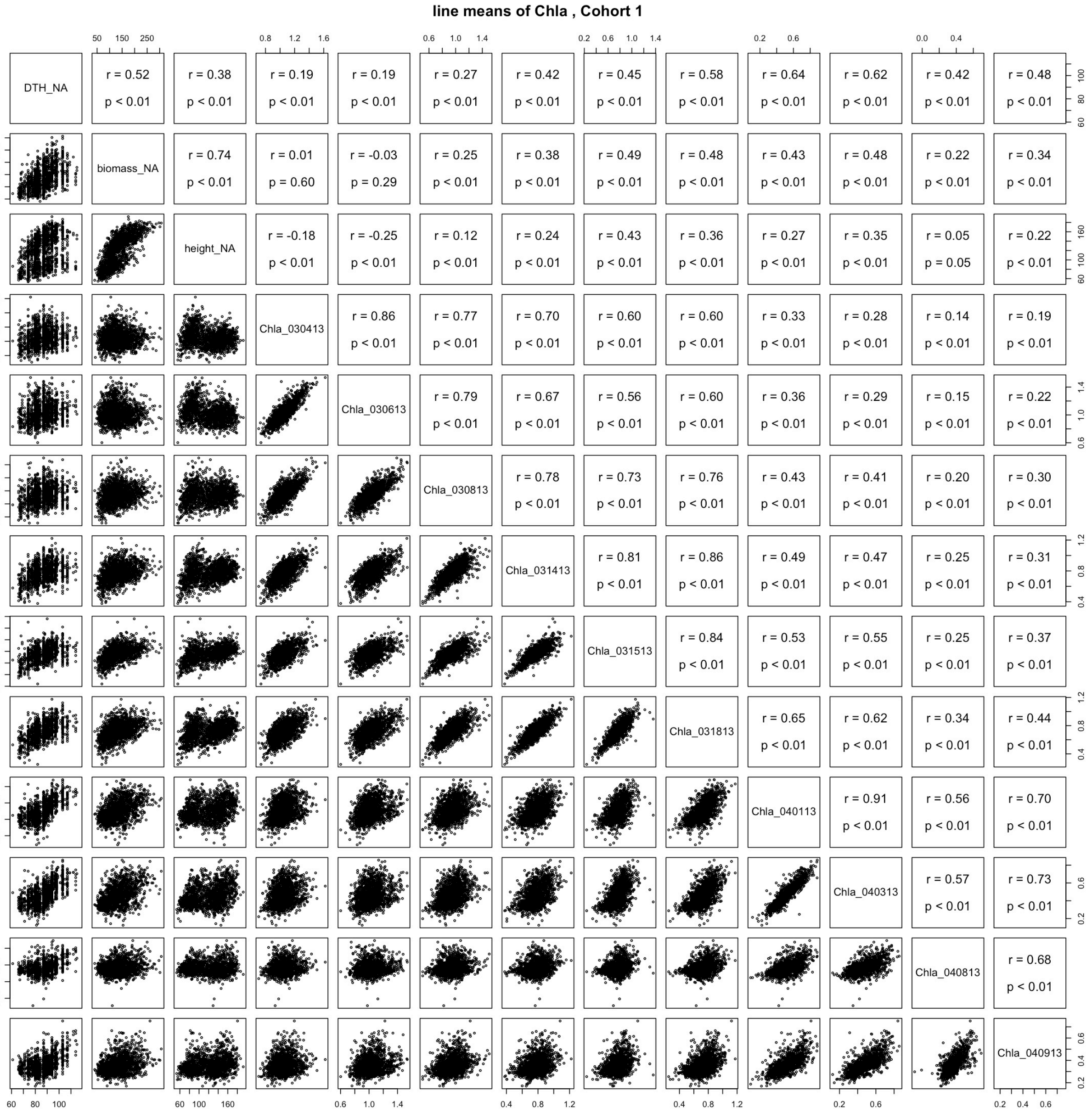
line means of Chla , Cohort 1
DTH_NA
biomass_NA
height_NA
Chla_030413
Chla_030613
Chla_030813
Chla_031413
Chla_031513
Chla_031813
Chla_040113
Chla_040313
Chla_040813
Chla_040913
r = 0.52 p < 0.01
r = 0.38 p < 0.01
r = 0.19 p < 0.01
r = 0.19 p < 0.01
r = 0.27 p < 0.01
r = 0.42 p < 0.01
r = 0.45 p < 0.01
r = 0.58 p < 0.01
r = 0.64 p < 0.01
r = 0.62 p < 0.01
r = 0.42 p < 0.01
r = 0.48 p < 0.01
r = 0.74 p < 0.01
r = 0.01 p = 0.60
r = -0.03 p = 0.29
r = 0.25 p < 0.01
r = 0.38 p < 0.01
r = 0.49 p < 0.01
r = 0.48 p < 0.01
r = 0.43 p < 0.01
r = 0.48 p < 0.01
r = 0.22 p < 0.01
r = 0.34 p < 0.01
r = -0.18 p < 0.01
r = -0.25 p < 0.01
r = 0.12 p < 0.01
r = 0.24 p < 0.01
r = 0.43 p < 0.01
r = 0.36 p < 0.01
r = 0.27 p < 0.01
r = 0.35 p < 0.01
r = 0.05 p = 0.05
r = 0.22 p < 0.01
r = 0.86 p < 0.01
r = 0.77 p < 0.01
r = 0.70 p < 0.01
r = 0.60 p < 0.01
r = 0.60 p < 0.01
r = 0.33 p < 0.01
r = 0.28 p < 0.01
r = 0.14 p < 0.01
r = 0.19 p < 0.01
r = 0.79 p < 0.01
r = 0.67 p < 0.01
r = 0.56 p < 0.01
r = 0.60 p < 0.01
r = 0.36 p < 0.01
r = 0.29 p < 0.01
r = 0.15 p < 0.01
r = 0.22 p < 0.01
r = 0.78 p < 0.01
r = 0.73 p < 0.01
r = 0.76 p < 0.01
r = 0.43 p < 0.01
r = 0.41 p < 0.01
r = 0.20 p < 0.01
r = 0.30 p < 0.01
r = 0.81 p < 0.01
r = 0.86 p < 0.01
r = 0.49 p < 0.01
r = 0.47 p < 0.01
r = 0.25 p < 0.01
r = 0.31 p < 0.01
r = 0.84 p < 0.01
r = 0.53 p < 0.01
r = 0.55 p < 0.01
r = 0.25 p < 0.01
r = 0.37 p < 0.01
r = 0.65 p < 0.01
r = 0.62 p < 0.01
r = 0.34 p < 0.01
r = 0.44 p < 0.01
r = 0.91 p < 0.01
r = 0.56 p < 0.01
r = 0.70 p < 0.01
r = 0.57 p < 0.01
r = 0.73 p < 0.01
r = 0.68 p < 0.01

Figure S2 B.

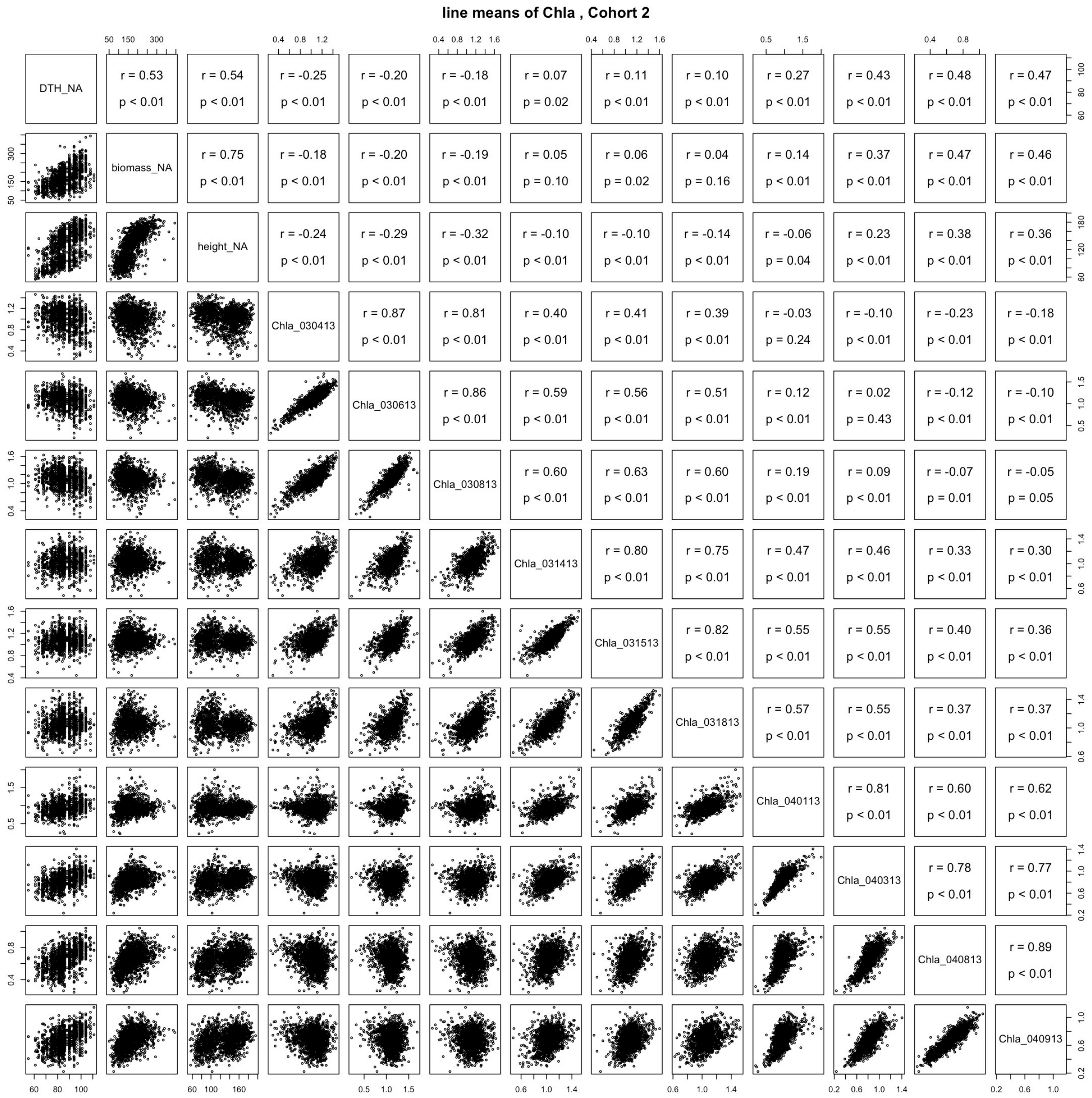

line means of Chla , Cohort 2
DTH_NA
biomass_NA
height_NA
Chla_030413
Chla_030613
Chla_030813
Chla_031413
Chla_031513
Chla_031813
Chla_040113
Chla_040313
Chla_040813
Chla_040913

Figure S2 C.

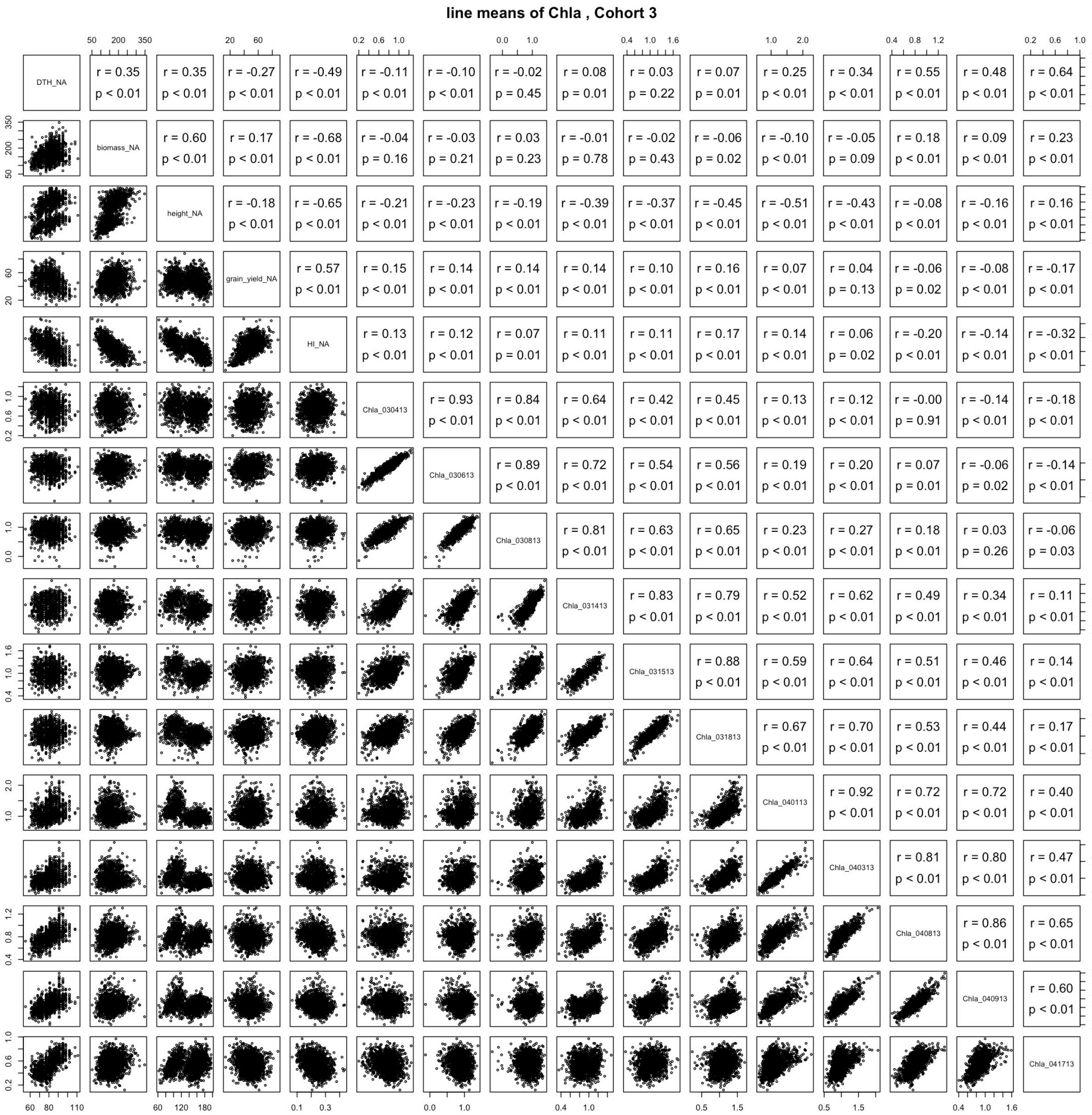
line means of Chla , Cohort 3
DTH_NA
biomass_NA
height_NA
grain_yield_NA
HI_NA
Chla_030413
Chla_030613
Chla_030813
Chla_031413
Chla_031513
Chla_031813
Chla_040113
Chla_040313
Chla_040813
Chla_040913
Chla_041713
r = 0.35 p < 0.01
r = 0.35 p < 0.01
r = -0.27 p < 0.01
r = -0.49 p < 0.01
r = -0.11 p < 0.01
r = -0.10 p < 0.01
r = -0.02 p = 0.45
r = 0.08 p = 0.01
r = 0.03 p = 0.22
r = 0.07 p = 0.01
r = 0.25 p < 0.01
r = 0.34 p < 0.01
r = 0.55 p < 0.01
r = 0.48 p < 0.01
r = 0.64 p < 0.01
r = 0.60 p < 0.01
r = 0.17 p < 0.01
r = -0.68 p < 0.01
r = -0.04 p = 0.16
r = -0.03 p = 0.21
r = 0.03 p = 0.23
r = -0.01 p = 0.78
r = -0.02 p = 0.43
r = -0.06 p = 0.02
r = -0.10 p < 0.01
r = -0.05 p = 0.09
r = 0.18 p < 0.01
r = 0.09 p < 0.01
r = 0.23 p < 0.01
r = -0.18 p < 0.01
r = -0.65 p < 0.01
r = -0.21 p < 0.01
r = -0.23 p < 0.01
r = -0.19 p < 0.01
r = -0.39 p < 0.01
r = -0.37 p < 0.01
r = -0.45 p < 0.01
r = -0.51 p < 0.01
r = -0.43 p < 0.01
r = -0.08 p < 0.01
r = -0.16 p < 0.01
r = 0.16 p < 0.01
r = 0.57 p < 0.01
r = 0.15 p < 0.01
r = 0.14 p < 0.01
r = 0.14 p < 0.01
r = 0.14 p < 0.01
r = 0.10 p < 0.01
r = 0.16 p < 0.01
r = 0.07 p < 0.01
r = 0.04 p = 0.13
r = -0.06 p = 0.02
r = -0.08 p < 0.01
r = -0.17 p < 0.01
r = 0.13 p < 0.01
r = 0.12 p < 0.01
r = 0.07 p = 0.01
r = 0.11 p < 0.01
r = 0.11 p < 0.01
r = 0.17 p < 0.01
r = 0.14 p < 0.01
r = 0.06 p = 0.02
r = -0.20 p < 0.01
r = -0.14 p < 0.01
r = -0.32 p < 0.01
r = 0.93 p < 0.01
r = 0.84 p < 0.01
r = 0.64 p < 0.01
r = 0.42 p < 0.01
r = 0.45 p < 0.01
r = 0.13 p < 0.01
r = 0.12 p < 0.01
r = -0.00 p = 0.91
r = -0.14 p < 0.01
r = -0.18 p < 0.01
r = 0.89 p < 0.01
r = 0.72 p < 0.01
r = 0.54 p < 0.01
r = 0.56 p < 0.01
r = 0.19 p < 0.01
r = 0.20 p < 0.01
r = 0.07 p = 0.01
r = -0.06 p = 0.02
r = -0.14 p < 0.01
r = 0.81 p < 0.01
r = 0.63 p < 0.01
r = 0.65 p < 0.01
r = 0.23 p < 0.01
r = 0.27 p < 0.01
r = 0.18 p < 0.01
r = 0.03 p = 0.26
r = -0.06 p = 0.03
r = 0.83 p < 0.01
r = 0.79 p < 0.01
r = 0.52 p < 0.01
r = 0.62 p < 0.01
r = 0.49 p < 0.01
r = 0.34 p < 0.01
r = 0.11 p < 0.01
r = 0.88 p < 0.01
r = 0.59 p < 0.01
r = 0.64 p < 0.01
r = 0.51 p < 0.01
r = 0.46 p < 0.01
r = 0.14 p < 0.01
r = 0.67 p < 0.01
r = 0.70 p < 0.01
r = 0.53 p < 0.01
r = 0.44 p < 0.01
r = 0.17 p < 0.01
r = 0.92 p < 0.01
r = 0.72 p < 0.01
r = 0.72 p < 0.01
r = 0.40 p < 0.01
r = 0.81 p < 0.01
r = 0.80 p < 0.01
r = 0.47 p < 0.01
r = 0.86 p < 0.01
r = 0.65 p < 0.01
r = 0.60 p < 0.01

Figure S2 D.

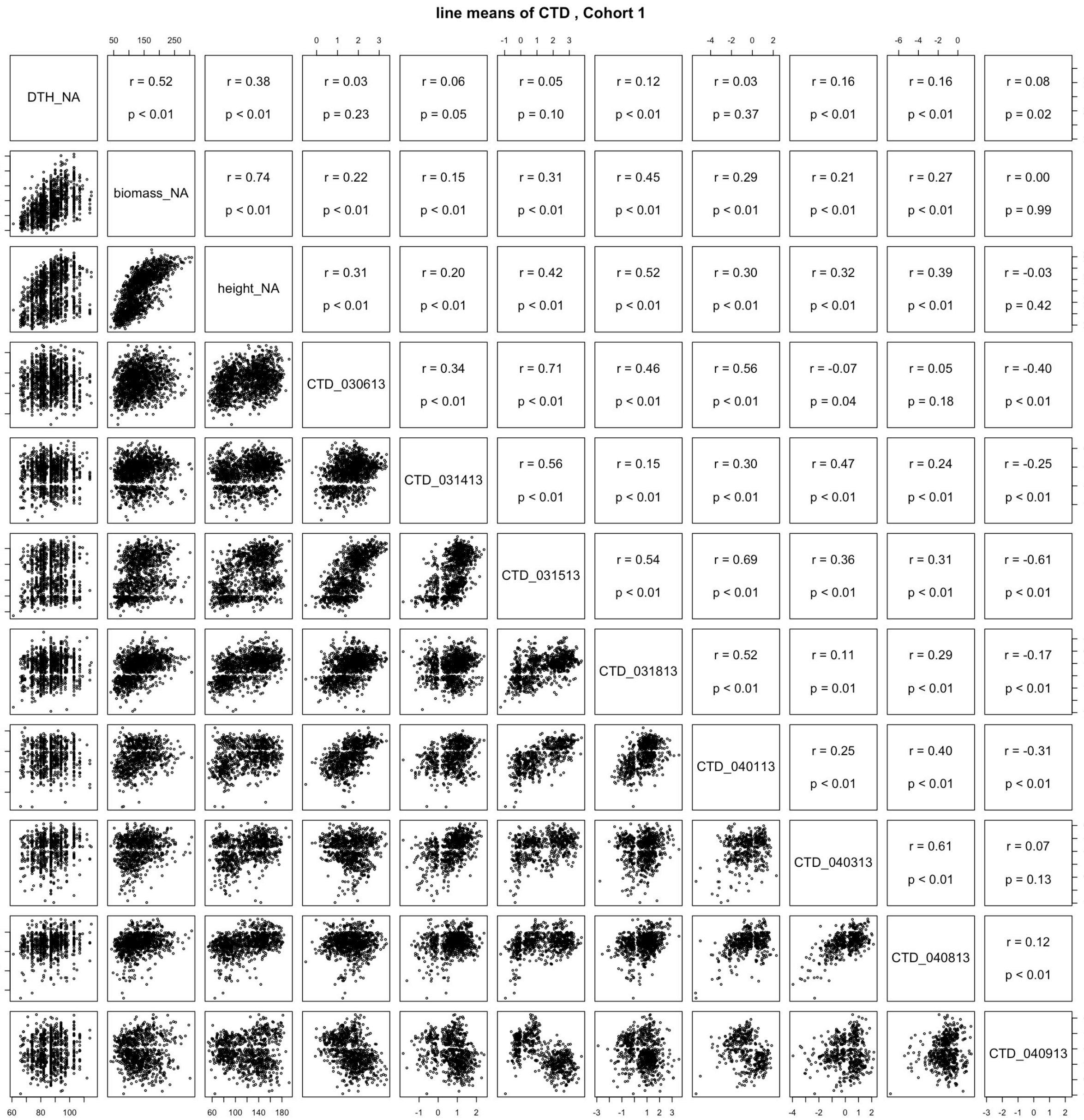
line means of CTD , Cohort 1
DTH_NA
biomass_NA
height_NA
CTD_030613
CTD_031413
CTD_031513
CTD_031813
CTD_040113
CTD_040313
CTD_040813
CTD_040913
r = 0.52 p < 0.01
r = 0.38 p < 0.01
r = 0.03 p = 0.23
r = 0.06 p = 0.05
r = 0.05 p = 0.10
r = 0.12 p < 0.01
r = 0.03 p = 0.37
r = 0.16 p < 0.01
r = 0.16 p < 0.01
r = 0.08 p = 0.02
r = 0.74 p < 0.01
r = 0.22 p < 0.01
r = 0.15 p < 0.01
r = 0.31 p < 0.01
r = 0.45 p < 0.01
r = 0.29 p < 0.01
r = 0.21 p < 0.01
r = 0.27 p < 0.01
r = 0.00 p = 0.99
r = 0.31 p < 0.01
r = 0.20 p < 0.01
r = 0.42 p < 0.01
r = 0.52 p < 0.01
r = 0.30 p < 0.01
r = 0.32 p < 0.01
r = 0.39 p < 0.01
r = -0.03 p = 0.42
r = 0.34 p < 0.01
r = 0.71 p < 0.01
r = 0.46 p < 0.01
r = 0.56 p < 0.01
r = -0.07 p = 0.04
r = 0.05 p = 0.18
r = -0.40 p < 0.01
r = 0.56 p < 0.01
r = 0.15 p < 0.01
r = 0.30 p < 0.01
r = 0.47 p < 0.01
r = 0.24 p < 0.01
r = -0.25 p < 0.01
r = 0.54 p < 0.01
r = 0.69 p < 0.01
r = 0.36 p < 0.01
r = 0.31 p < 0.01
r = -0.61 p < 0.01
r = 0.52 p < 0.01
r = 0.11 p = 0.01
r = 0.29 p < 0.01
r = -0.17 p < 0.01
r = 0.25 p < 0.01
r = 0.40 p < 0.01
r = -0.31 p < 0.01
r = 0.61 p < 0.01
r = 0.07 p = 0.13
r = 0.12 p < 0.01

Figure S2 E.

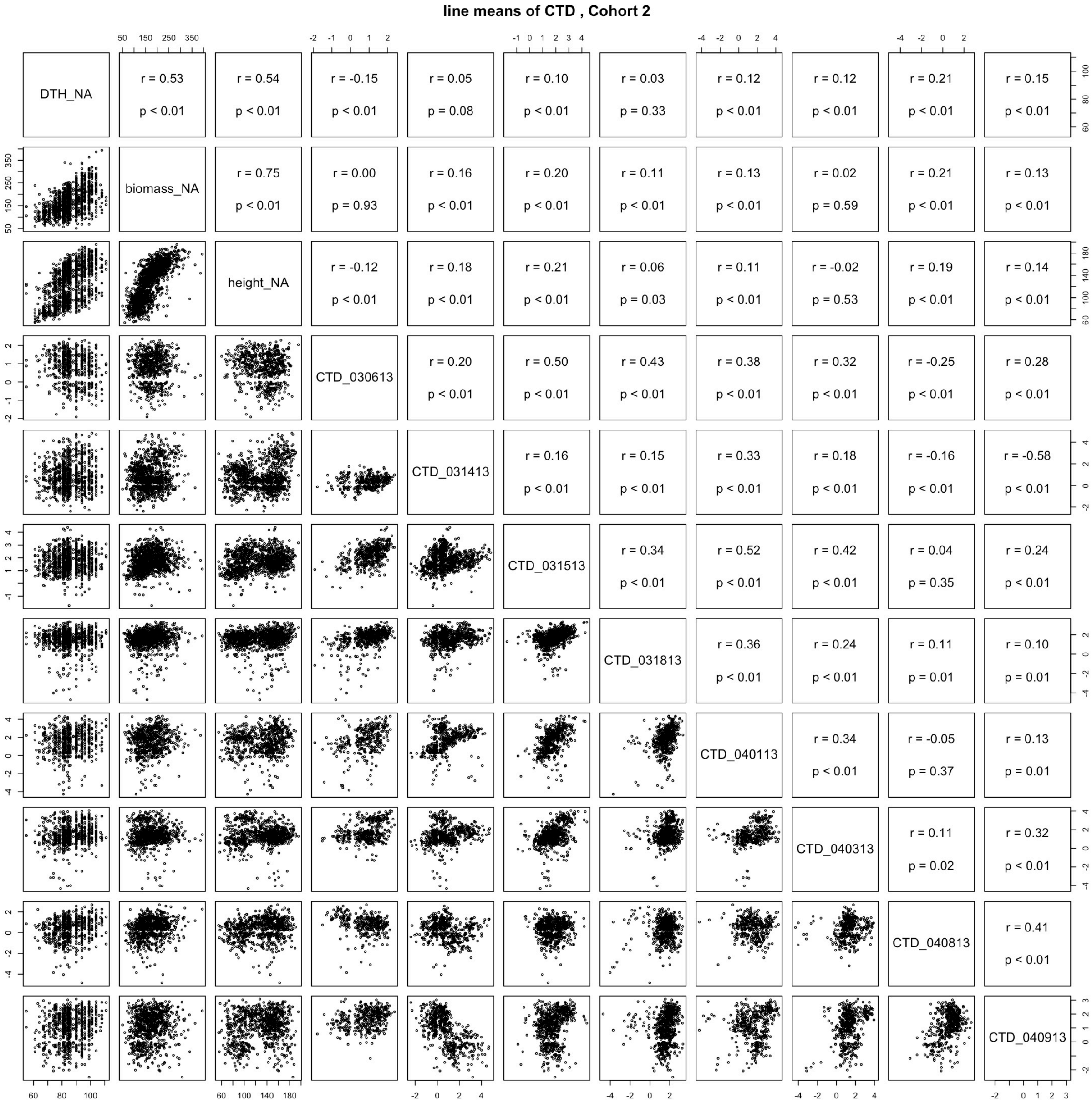
line means of CTD , Cohort 2
DTH_NA
biomass_NA
height_NA
CTD_030613
CTD_031413
CTD_031513
CTD_031813
CTD_040113
CTD_040313
CTD_040813
CTD_040913
r = 0.53 p < 0.01
r = 0.54 p < 0.01
r = -0.15 p < 0.01
r = 0.05 p = 0.08
r = 0.10 p < 0.01
r = 0.03 p = 0.33
r = 0.12 p < 0.01
r = 0.12 p < 0.01
r = 0.21 p < 0.01
r = 0.15 p < 0.01
r = 0.75 p < 0.01
r = 0.00 p = 0.93
r = 0.16 p < 0.01
r = 0.20 p < 0.01
r = 0.11 p < 0.01
r = 0.13 p < 0.01
r = 0.02 p = 0.59
r = 0.21 p < 0.01
r = 0.13 p < 0.01
r = -0.12 p < 0.01
r = 0.18 p < 0.01
r = 0.21 p < 0.01
r = 0.06 p = 0.03
r = 0.11 p < 0.01
r = -0.02 p = 0.53
r = 0.19 p < 0.01
r = 0.14 p < 0.01
r = 0.20 p < 0.01
r = 0.50 p < 0.01
r = 0.43 p < 0.01
r = 0.38 p < 0.01
r = 0.32 p < 0.01
r = -0.25 p < 0.01
r = 0.28 p < 0.01
r = 0.16 p < 0.01
r = 0.15 p < 0.01
r = 0.33 p < 0.01
r = 0.18 p < 0.01
r = -0.16 p < 0.01
r = -0.58 p < 0.01
r = 0.34 p < 0.01
r = 0.52 p < 0.01
r = 0.42 p < 0.01
r = 0.04 p = 0.35
r = 0.24 p < 0.01
r = 0.36 p < 0.01
r = 0.24 p < 0.01
r = 0.11 p = 0.01
r = 0.10 p = 0.01
r = 0.34 p < 0.01
r = -0.05 p = 0.37
r = 0.13 p = 0.01
r = 0.11 p = 0.02
r = 0.32 p < 0.01
r = 0.41 p < 0.01

Figure S2 F.

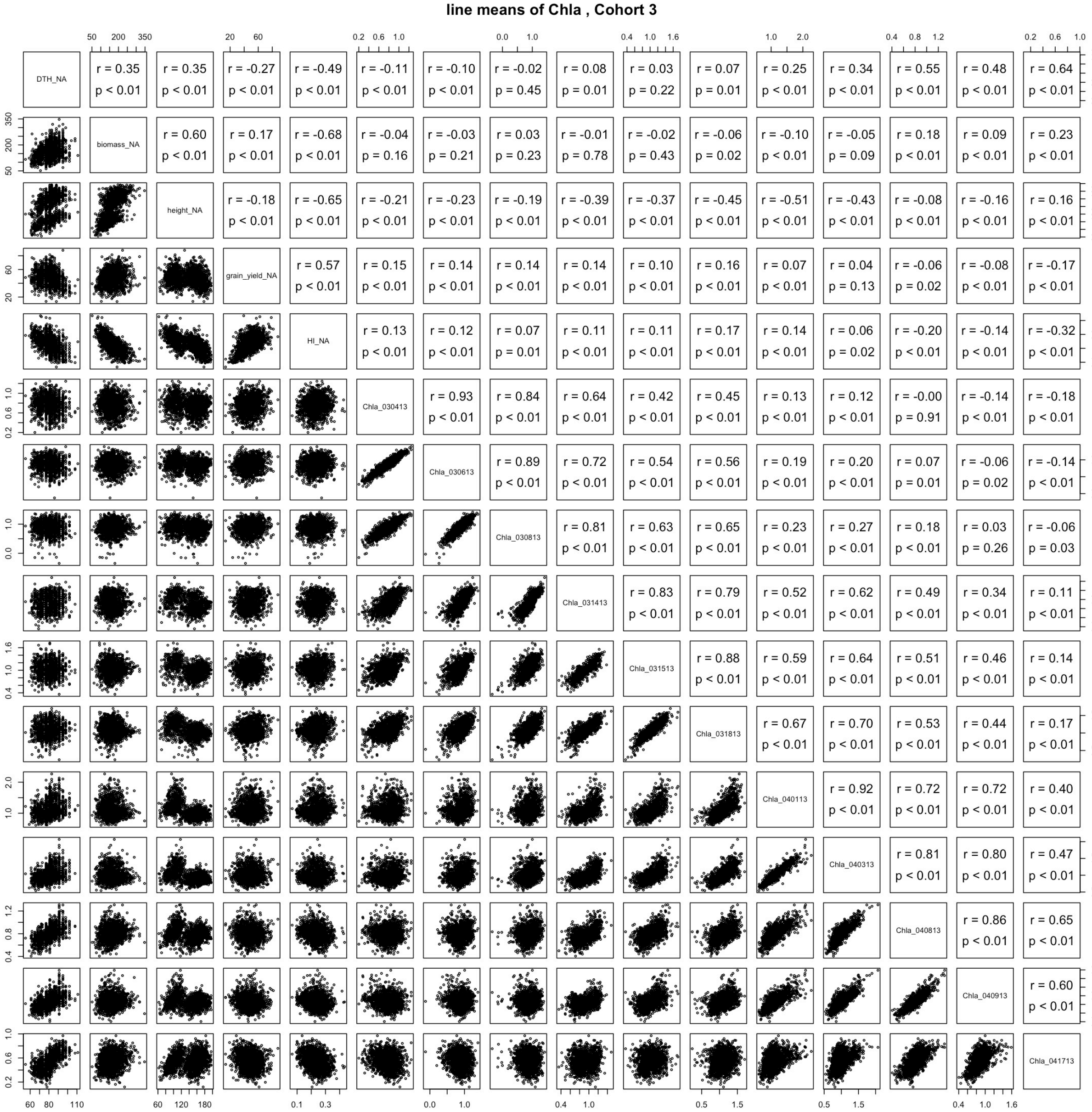
line means of Chla , Cohort 3
DTH_NA
biomass_NA
height_NA
grain_yield_NA
HI_NA
Chla_030413
Chla_030613
Chla_030813
Chla_031413
Chla_031513
Chla_031813
Chla_040113
Chla_040313
Chla_040813
Chla_040913
Chla_041713

Figure S2 G.

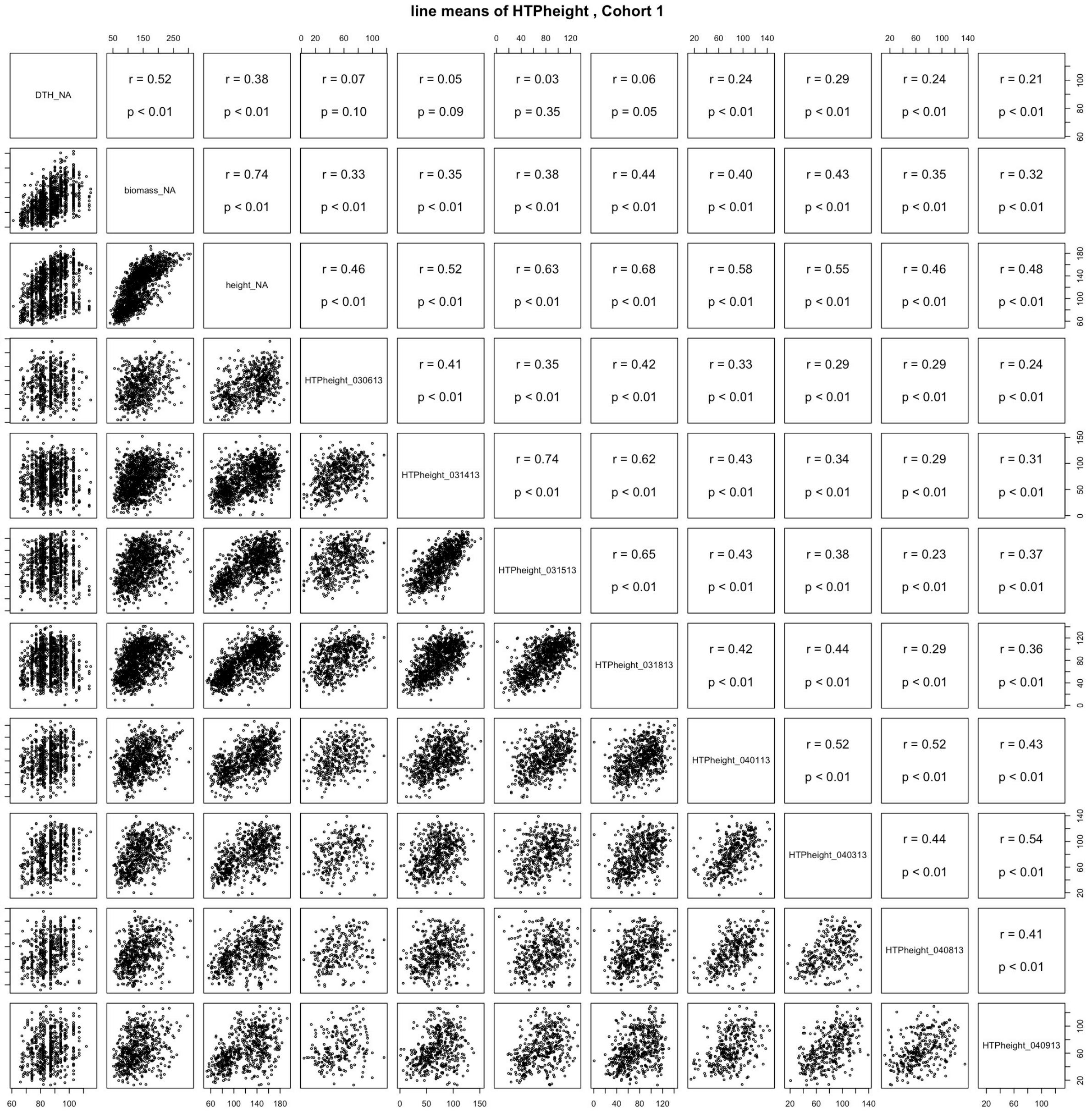
line means of HTPheight , Cohort 1
DTH_NA
biomass_NA
height_NA
HTPheight_030613
HTPheight_031413
HTPheight_031513
HTPheight_031813
HTPheight_040113
HTPheight_040313
HTPheight_040813
HTPheight_040913
r = 0.52 p < 0.01
r = 0.38 p < 0.01
r = 0.07 p = 0.10
r = 0.05 p = 0.09
r = 0.03 p = 0.35
r = 0.06 p = 0.05
r = 0.24 p < 0.01
r = 0.29 p < 0.01
r = 0.24 p < 0.01
r = 0.21 p < 0.01
r = 0.74 p < 0.01
r = 0.33 p < 0.01
r = 0.35 p < 0.01
r = 0.38 p < 0.01
r = 0.44 p < 0.01
r = 0.40 p < 0.01
r = 0.43 p < 0.01
r = 0.35 p < 0.01
r = 0.32 p < 0.01
r = 0.46 p < 0.01
r = 0.52 p < 0.01
r = 0.63 p < 0.01
r = 0.68 p < 0.01
r = 0.58 p < 0.01
r = 0.55 p < 0.01
r = 0.46 p < 0.01
r = 0.48 p < 0.01
r = 0.41 p < 0.01
r = 0.35 p < 0.01
r = 0.42 p < 0.01
r = 0.33 p < 0.01
r = 0.29 p < 0.01
r = 0.29 p < 0.01
r = 0.24 p < 0.01
r = 0.74 p < 0.01
r = 0.62 p < 0.01
r = 0.43 p < 0.01
r = 0.34 p < 0.01
r = 0.29 p < 0.01
r = 0.31 p < 0.01
r = 0.65 p < 0.01
r = 0.43 p < 0.01
r = 0.38 p < 0.01
r = 0.23 p < 0.01
r = 0.37 p < 0.01
r = 0.42 p < 0.01
r = 0.44 p < 0.01
r = 0.29 p < 0.01
r = 0.36 p < 0.01
r = 0.52 p < 0.01
r = 0.52 p < 0.01
r = 0.43 p < 0.01
r = 0.44 p < 0.01
r = 0.54 p < 0.01
r = 0.41 p < 0.01

Figure S2 H.

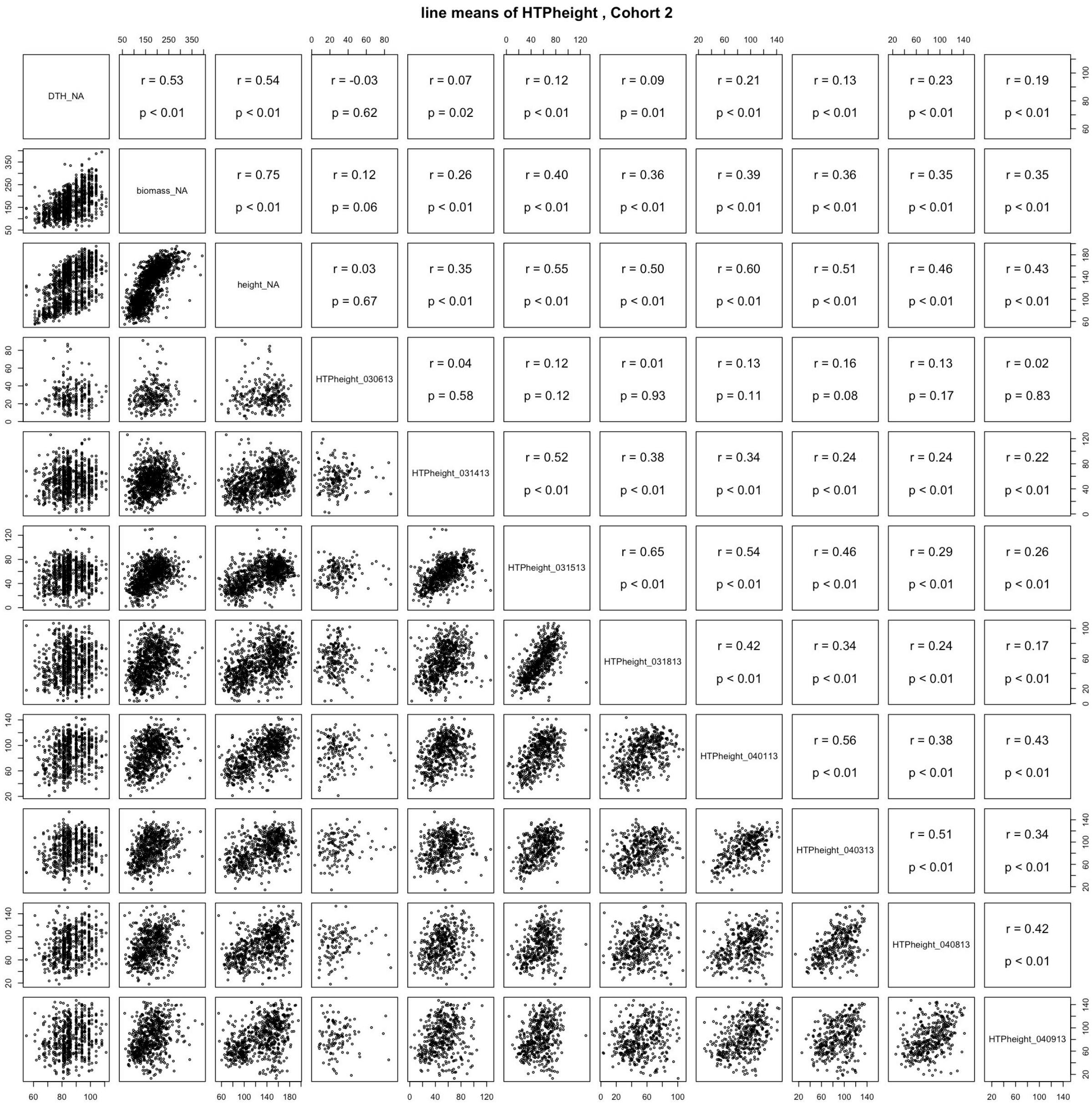
line means of HTPheight , Cohort 2
DTH_NA
biomass_NA
height_NA
HTPheight_030613
HTPheight_031413
HTPheight_031513
HTPheight_031813
HTPheight_040113
HTPheight_040313
HTPheight_040813
HTPheight_040913
r = 0.53 p < 0.01
r = 0.54 p < 0.01
r = -0.03 p = 0.62
r = 0.07 p = 0.02
r = 0.12 p < 0.01
r = 0.09 p = 0.01
r = 0.21 p < 0.01
r = 0.13 p < 0.01
r = 0.23 p < 0.01
r = 0.19 p < 0.01
r = 0.75 p < 0.01
r = 0.12 p = 0.06
r = 0.26 p < 0.01
r = 0.40 p < 0.01
r = 0.36 p < 0.01
r = 0.39 p < 0.01
r = 0.36 p < 0.01
r = 0.35 p < 0.01
r = 0.35 p < 0.01
r = 0.03 p = 0.67
r = 0.35 p < 0.01
r = 0.55 p < 0.01
r = 0.50 p < 0.01
r = 0.60 p < 0.01
r = 0.51 p < 0.01
r = 0.46 p < 0.01
r = 0.43 p < 0.01
r = 0.04 p = 0.58
r = 0.12 p = 0.12
r = 0.01 p = 0.93
r = 0.13 p = 0.11
r = 0.16 p = 0.08
r = 0.13 p = 0.17
r = 0.02 p = 0.83
r = 0.52 p < 0.01
r = 0.38 p < 0.01
r = 0.34 p < 0.01
r = 0.24 p < 0.01
r = 0.24 p < 0.01
r = 0.22 p < 0.01
r = 0.65 p < 0.01
r = 0.54 p < 0.01
r = 0.46 p < 0.01
r = 0.29 p < 0.01
r = 0.26 p < 0.01
r = 0.42 p < 0.01
r = 0.34 p < 0.01
r = 0.24 p < 0.01
r = 0.17 p < 0.01
r = 0.56 p < 0.01
r = 0.38 p < 0.01
r = 0.43 p < 0.01
r = 0.51 p < 0.01
r = 0.34 p < 0.01
r = 0.42 p < 0.01

Figure S2 I.

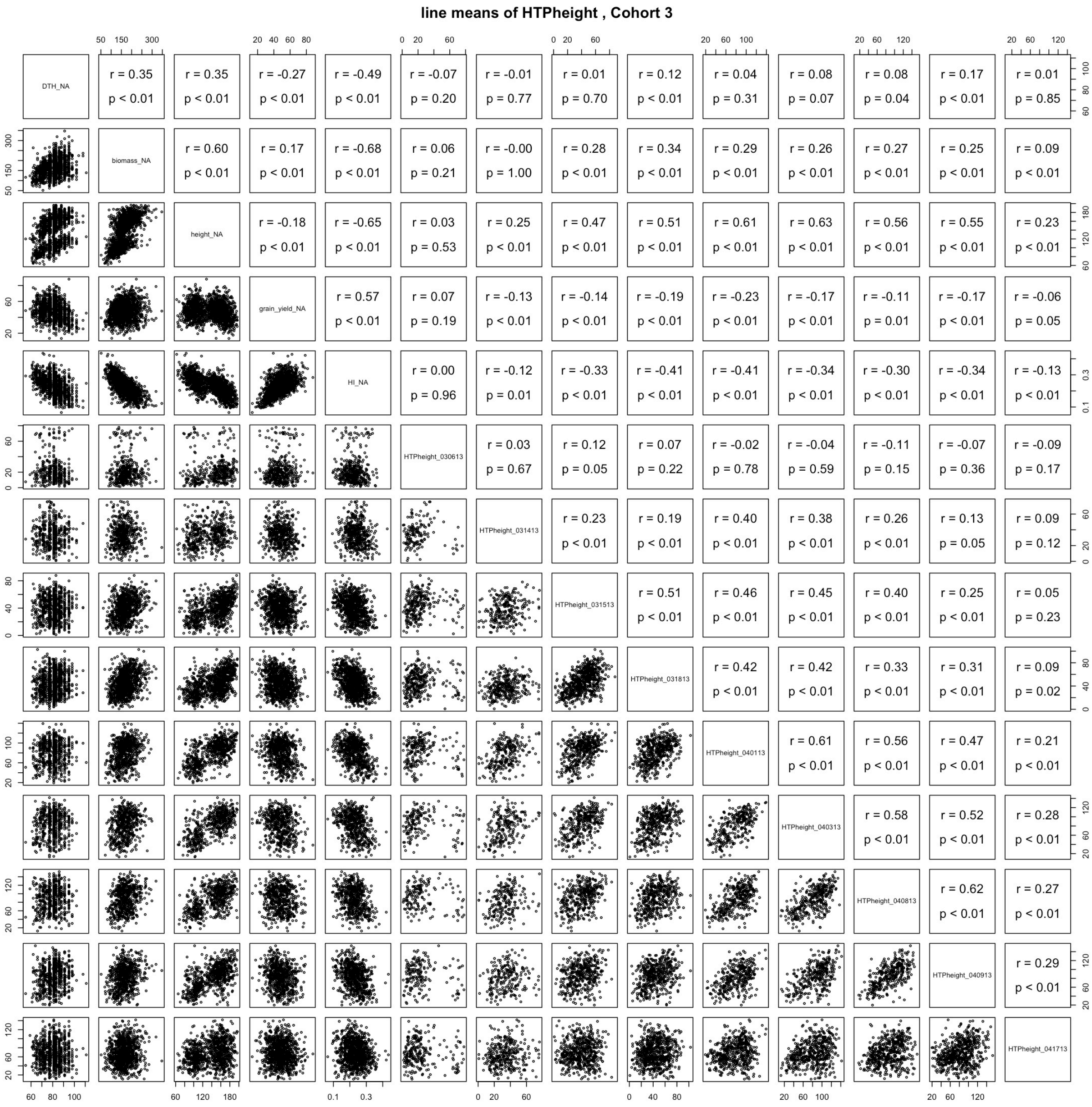
line means of HTPheight , Cohort 3
DTH_NA
r = 0.35 p < 0.01
r = 0.35 p < 0.01
r = -0.27 p < 0.01
r = -0.49 p < 0.01
r = -0.07 p = 0.20
r = -0.01 p = 0.77
r = 0.01 p = 0.70
r = 0.12 p < 0.01
r = 0.04 p = 0.31
r = 0.08 p = 0.07
r = 0.08 p = 0.04
r = 0.17 p < 0.01
r = 0.01 p = 0.85
biomass_NA
r = 0.60 p < 0.01
r = 0.17 p < 0.01
r = -0.68 p < 0.01
r = 0.06 p = 0.21
r = -0.00 p = 1.00
r = 0.28 p < 0.01
r = 0.34 p < 0.01
r = 0.29 p < 0.01
r = 0.26 p < 0.01
r = 0.27 p < 0.01
r = 0.25 p < 0.01
r = 0.09 p < 0.01
height_NA
r = -0.18 p < 0.01
r = -0.65 p < 0.01
r = 0.03 p = 0.53
r = 0.25 p < 0.01
r = 0.47 p < 0.01
r = 0.51 p < 0.01
r = 0.61 p < 0.01
r = 0.63 p < 0.01
r = 0.56 p < 0.01
r = 0.55 p < 0.01
r = 0.23 p < 0.01
grain_yield_NA
r = 0.57 p < 0.01
r = 0.07 p = 0.19
r = -0.13 p < 0.01
r = -0.14 p < 0.01
r = -0.19 p < 0.01
r = -0.23 p < 0.01
r = -0.17 p < 0.01
r = -0.11 p = 0.01
r = -0.17 p < 0.01
r = -0.06 p = 0.05
HI_NA
r = 0.00 p = 0.96
r = -0.12 p = 0.01
r = -0.33 p < 0.01
r = -0.41 p < 0.01
r = -0.41 p < 0.01
r = -0.34 p < 0.01
r = -0.30 p < 0.01
r = -0.34 p < 0.01
r = -0.13 p < 0.01
HTPheight_030613
r = 0.03 p = 0.67
r = 0.12 p = 0.05
r = 0.07 p = 0.22
r = -0.02 p = 0.78
r = -0.04 p = 0.59
r = -0.11 p = 0.15
r = -0.07 p = 0.36
r = -0.09 p = 0.17
HTPheight_031413
r = 0.23 p < 0.01
r = 0.19 p < 0.01
r = 0.40 p < 0.01
r = 0.38 p < 0.01
r = 0.26 p < 0.01
r = 0.13 p = 0.05
r = 0.09 p = 0.12
HTPheight_031513
r = 0.51 p < 0.01
r = 0.46 p < 0.01
r = 0.45 p < 0.01
r = 0.40 p < 0.01
r = 0.25 p < 0.01
r = 0.05 p = 0.23
HTPheight_031813
r = 0.42 p < 0.01
r = 0.42 p < 0.01
r = 0.33 p < 0.01
r = 0.31 p < 0.01
r = 0.09 p = 0.02
HTPheight_040113
r = 0.61 p < 0.01
r = 0.56 p < 0.01
r = 0.47 p < 0.01
r = 0.21 p < 0.01
HTPheight_040313
r = 0.58 p < 0.01
r = 0.52 p < 0.01
r = 0.28 p < 0.01
HTPheight_040813
r = 0.62 p < 0.01
r = 0.27 p < 0.01
HTPheight_040913
r = 0.29 p < 0.01
HTPheight_041713

Figure S2 J.

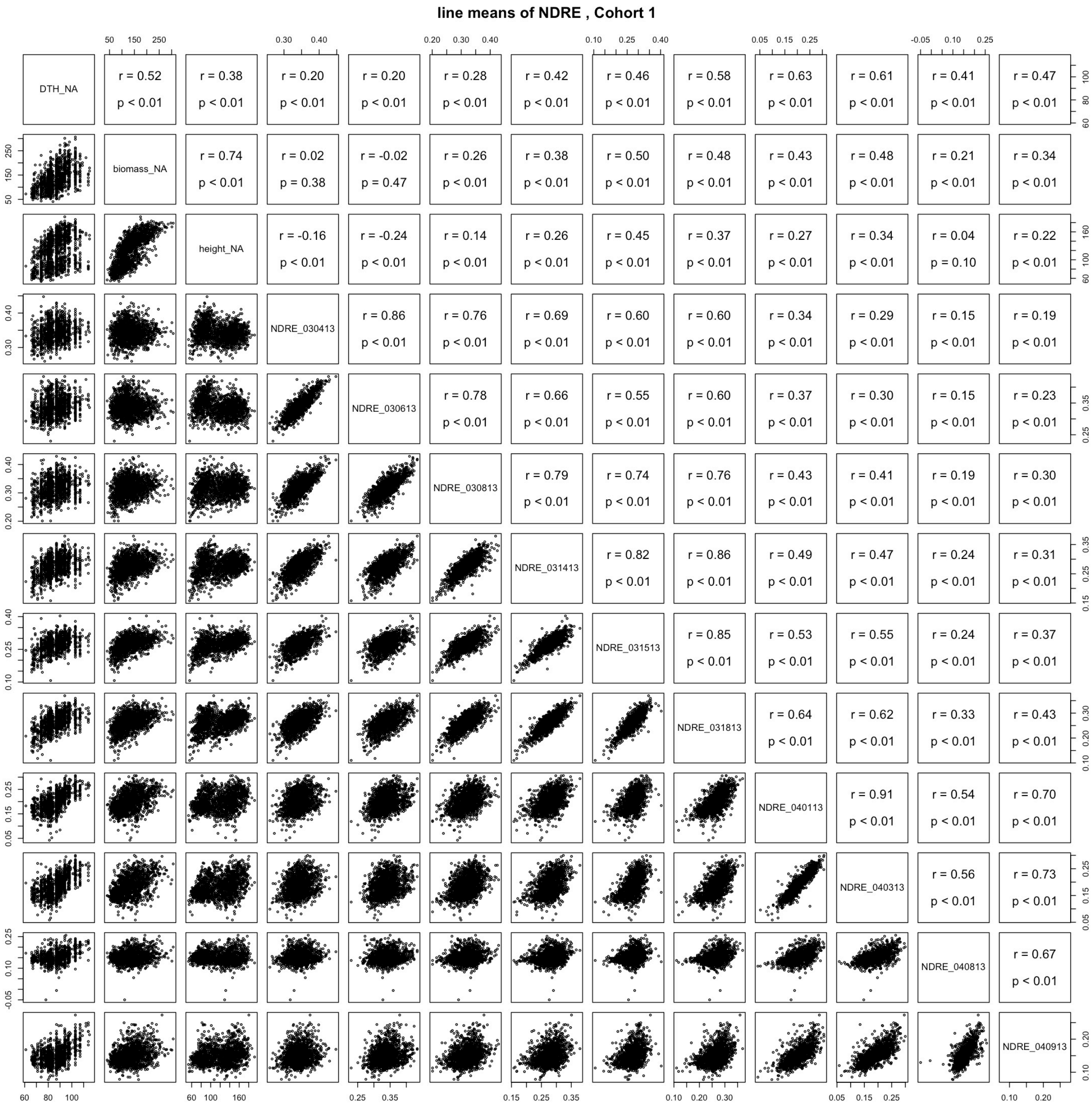
line means of NDRE , Cohort 1
DTH_NA
r = 0.52 p < 0.01
r = 0.38 p < 0.01
r = 0.20 p < 0.01
r = 0.20 p < 0.01
r = 0.28 p < 0.01
r = 0.42 p < 0.01
r = 0.46 p < 0.01
r = 0.58 p < 0.01
r = 0.63 p < 0.01
r = 0.61 p < 0.01
r = 0.41 p < 0.01
r = 0.47 p < 0.01
biomass_NA
r = 0.74 p < 0.01
r = 0.02 p = 0.38
r = -0.02 p = 0.47
r = 0.26 p < 0.01
r = 0.38 p < 0.01
r = 0.50 p < 0.01
r = 0.48 p < 0.01
r = 0.43 p < 0.01
r = 0.48 p < 0.01
r = 0.21 p < 0.01
r = 0.34 p < 0.01
height_NA
r = -0.16 p < 0.01
r = -0.24 p < 0.01
r = 0.14 p < 0.01
r = 0.26 p < 0.01
r = 0.45 p < 0.01
r = 0.37 p < 0.01
r = 0.27 p < 0.01
r = 0.34 p < 0.01
r = 0.04 p = 0.10
r = 0.22 p < 0.01
NDRE_030413
r = 0.86 p < 0.01
r = 0.76 p < 0.01
r = 0.69 p < 0.01
r = 0.60 p < 0.01
r = 0.60 p < 0.01
r = 0.34 p < 0.01
r = 0.29 p < 0.01
r = 0.15 p < 0.01
r = 0.19 p < 0.01
NDRE_030613
r = 0.78 p < 0.01
r = 0.66 p < 0.01
r = 0.55 p < 0.01
r = 0.60 p < 0.01
r = 0.37 p < 0.01
r = 0.30 p < 0.01
r = 0.15 p < 0.01
r = 0.23 p < 0.01
NDRE_030813
r = 0.79 p < 0.01
r = 0.74 p < 0.01
r = 0.76 p < 0.01
r = 0.43 p < 0.01
r = 0.41 p < 0.01
r = 0.19 p < 0.01
r = 0.30 p < 0.01
NDRE_031413
r = 0.82 p < 0.01
r = 0.86 p < 0.01
r = 0.49 p < 0.01
r = 0.47 p < 0.01
r = 0.24 p < 0.01
r = 0.31 p < 0.01
NDRE_031513
r = 0.85 p < 0.01
r = 0.53 p < 0.01
r = 0.55 p < 0.01
r = 0.24 p < 0.01
r = 0.37 p < 0.01
NDRE_031813
r = 0.64 p < 0.01
r = 0.62 p < 0.01
r = 0.33 p < 0.01
r = 0.43 p < 0.01
NDRE_040113
r = 0.91 p < 0.01
r = 0.54 p < 0.01
r = 0.70 p < 0.01
NDRE_040313
r = 0.56 p < 0.01
r = 0.73 p < 0.01
NDRE_040813
r = 0.67 p < 0.01
NDRE_040913

Figure S2 K.

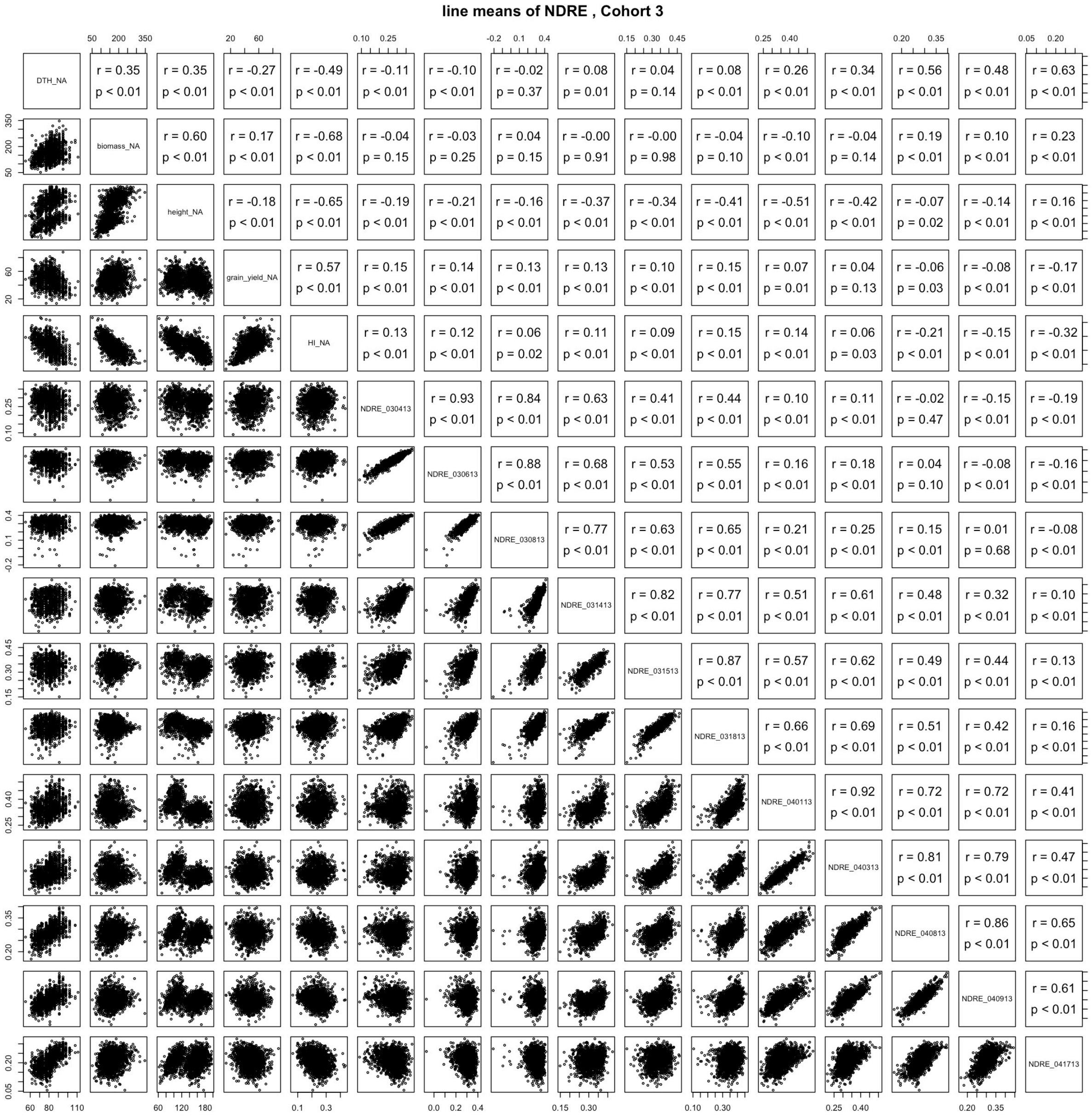
line means of NDRE , Cohort 3
DTH_NA
r = 0.35 p < 0.01
r = 0.35 p < 0.01
r = -0.27 p < 0.01
r = -0.49 p < 0.01
r = -0.11 p < 0.01
r = -0.10 p < 0.01
r = -0.02 p = 0.37
r = 0.08 p = 0.01
r = 0.04 p = 0.14
r = 0.08 p < 0.01
r = 0.26 p < 0.01
r = 0.34 p < 0.01
r = 0.56 p < 0.01
r = 0.48 p < 0.01
r = 0.63 p < 0.01
biomass_NA
r = 0.60 p < 0.01
r = 0.17 p < 0.01
r = -0.68 p < 0.01
r = -0.04 p = 0.15
r = -0.03 p = 0.25
r = 0.04 p = 0.15
r = -0.00 p = 0.91
r = -0.00 p = 0.98
r = -0.04 p = 0.10
r = -0.10 p < 0.01
r = -0.04 p = 0.14
r = 0.19 p < 0.01
r = 0.10 p < 0.01
r = 0.23 p < 0.01
height_NA
r = -0.18 p < 0.01
r = -0.65 p < 0.01
r = -0.19 p < 0.01
r = -0.21 p < 0.01
r = -0.16 p < 0.01
r = -0.37 p < 0.01
r = -0.34 p < 0.01
r = -0.41 p < 0.01
r = -0.51 p < 0.01
r = -0.42 p < 0.01
r = -0.07 p = 0.02
r = -0.14 p < 0.01
r = 0.16 p < 0.01
grain_yield_NA
r = 0.57 p < 0.01
r = 0.15 p < 0.01
r = 0.14 p < 0.01
r = 0.13 p < 0.01
r = 0.13 p < 0.01
r = 0.10 p < 0.01
r = 0.15 p < 0.01
r = 0.07 p = 0.01
r = 0.04 p = 0.13
r = -0.06 p = 0.03
r = -0.08 p < 0.01
r = -0.17 p < 0.01
HI_NA
r = 0.13 p < 0.01
r = 0.12 p < 0.01
r = 0.06 p = 0.02
r = 0.11 p < 0.01
r = 0.09 p < 0.01
r = 0.15 p < 0.01
r = 0.14 p < 0.01
r = 0.06 p = 0.03
r = -0.21 p < 0.01
r = -0.15 p < 0.01
r = -0.32 p < 0.01
NDRE_030413
r = 0.93 p < 0.01
r = 0.84 p < 0.01
r = 0.63 p < 0.01
r = 0.41 p < 0.01
r = 0.44 p < 0.01
r = 0.10 p < 0.01
r = 0.11 p < 0.01
r = -0.02 p = 0.47
r = -0.15 p < 0.01
r = -0.19 p < 0.01
NDRE_030613
r = 0.88 p < 0.01
r = 0.68 p < 0.01
r = 0.53 p < 0.01
r = 0.55 p < 0.01
r = 0.16 p < 0.01
r = 0.18 p < 0.01
r = 0.04 p = 0.10
r = -0.08 p < 0.01
r = -0.16 p < 0.01
NDRE_030813
r = 0.77 p < 0.01
r = 0.63 p < 0.01
r = 0.65 p < 0.01
r = 0.21 p < 0.01
r = 0.25 p < 0.01
r = 0.15 p < 0.01
r = 0.01 p = 0.68
r = -0.08 p < 0.01
NDRE_031413
r = 0.82 p < 0.01
r = 0.77 p < 0.01
r = 0.51 p < 0.01
r = 0.61 p < 0.01
r = 0.48 p < 0.01
r = 0.32 p < 0.01
r = 0.10 p < 0.01
NDRE_031513
r = 0.87 p < 0.01
r = 0.57 p < 0.01
r = 0.62 p < 0.01
r = 0.49 p < 0.01
r = 0.44 p < 0.01
r = 0.13 p < 0.01
NDRE_031813
r = 0.66 p < 0.01
r = 0.69 p < 0.01
r = 0.51 p < 0.01
r = 0.42 p < 0.01
r = 0.16 p < 0.01
NDRE_040113
r = 0.92 p < 0.01
r = 0.72 p < 0.01
r = 0.72 p < 0.01
r = 0.41 p < 0.01
NDRE_040313
r = 0.81 p < 0.01
r = 0.79 p < 0.01
r = 0.47 p < 0.01
NDRE_040813
r = 0.86 p < 0.01
r = 0.65 p < 0.01
NDRE_040913
r = 0.61 p < 0.01
NDRE_041713

Figure S2 L.

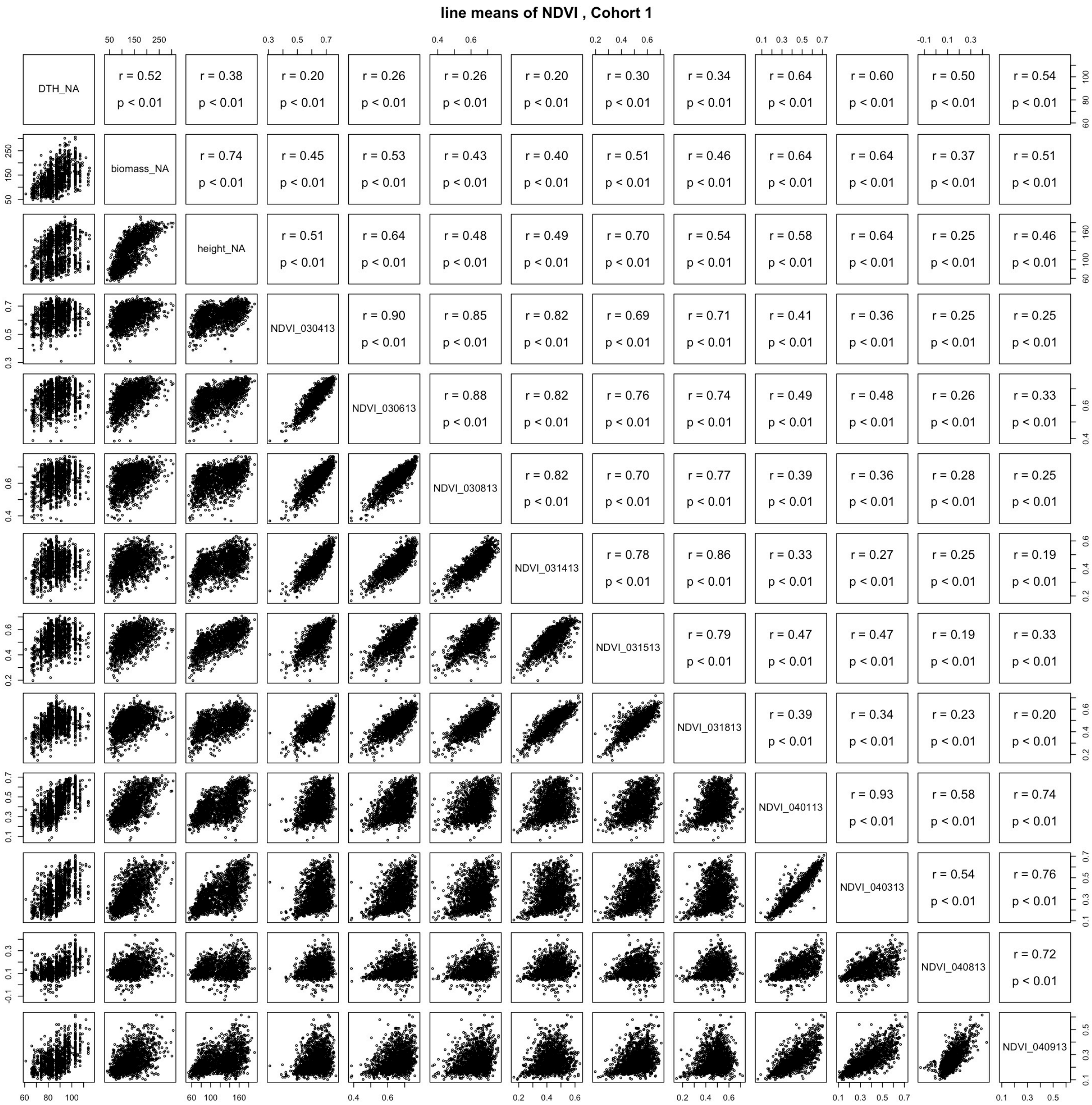
line means of NDVI , Cohort 1
DTH_NA
biomass_NA
height_NA
NDVI_030413
NDVI_030613
NDVI_030813
NDVI_031413
NDVI_031513
NDVI_031813
NDVI_040113
NDVI_040313
NDVI_040813
NDVI_040913

Figure S2 M.

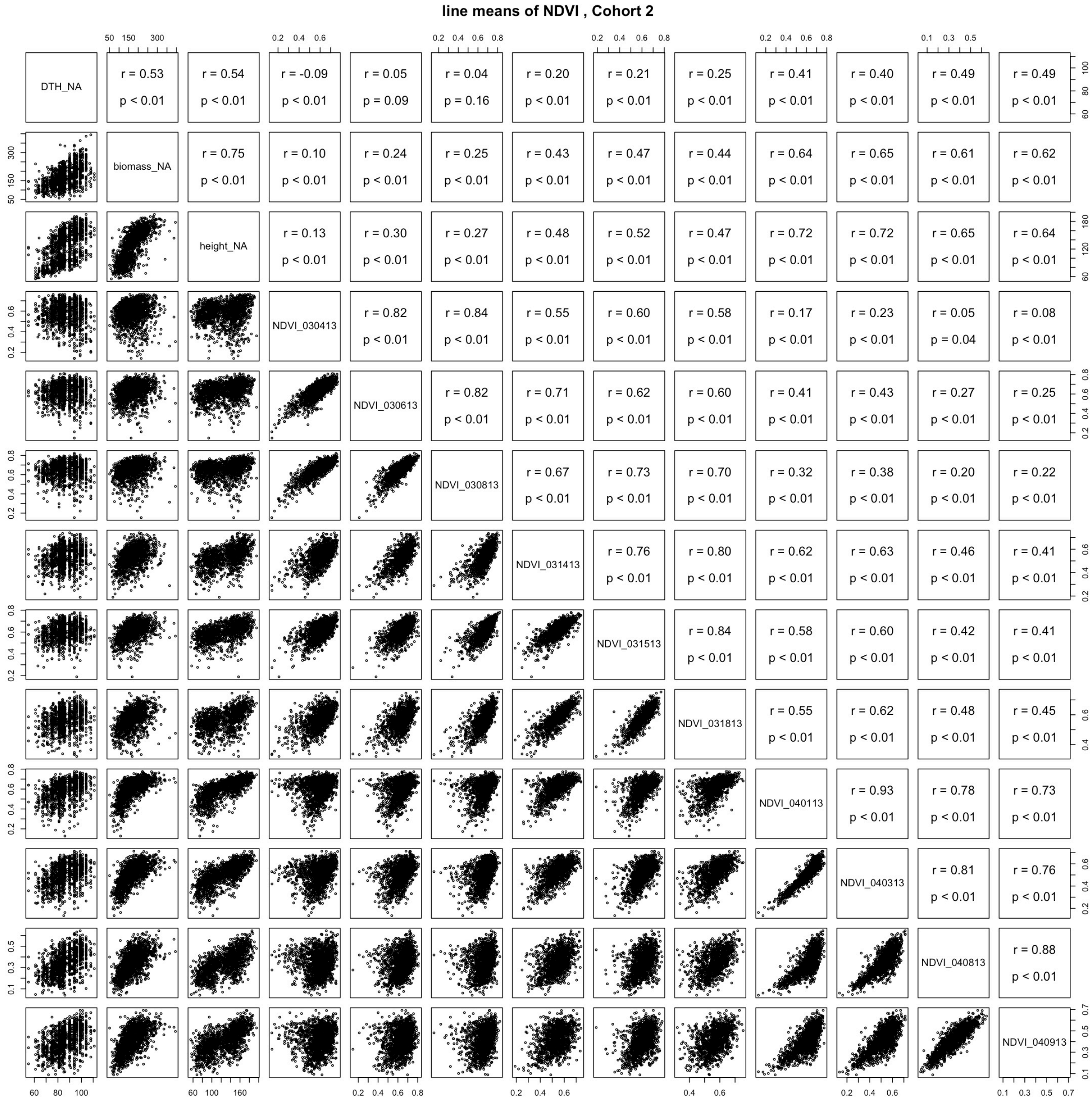
line means of NDVI , Cohort 2
DTH_NA
biomass_NA
height_NA
NDVI_030413
NDVI_030613
NDVI_030813
NDVI_031413
NDVI_031513
NDVI_031813
NDVI_040113
NDVI_040313
NDVI_040813
NDVI_040913
r = 0.53 p < 0.01
r = 0.54 p < 0.01
r = -0.09 p < 0.01
r = 0.05 p = 0.09
r = 0.04 p = 0.16
r = 0.20 p < 0.01
r = 0.21 p < 0.01
r = 0.25 p < 0.01
r = 0.41 p < 0.01
r = 0.40 p < 0.01
r = 0.49 p < 0.01
r = 0.49 p < 0.01
r = 0.75 p < 0.01
r = 0.10 p < 0.01
r = 0.24 p < 0.01
r = 0.25 p < 0.01
r = 0.43 p < 0.01
r = 0.47 p < 0.01
r = 0.44 p < 0.01
r = 0.64 p < 0.01
r = 0.65 p < 0.01
r = 0.61 p < 0.01
r = 0.62 p < 0.01
r = 0.13 p < 0.01
r = 0.30 p < 0.01
r = 0.27 p < 0.01
r = 0.48 p < 0.01
r = 0.52 p < 0.01
r = 0.47 p < 0.01
r = 0.72 p < 0.01
r = 0.72 p < 0.01
r = 0.65 p < 0.01
r = 0.64 p < 0.01
r = 0.82 p < 0.01
r = 0.84 p < 0.01
r = 0.55 p < 0.01
r = 0.60 p < 0.01
r = 0.58 p < 0.01
r = 0.17 p < 0.01
r = 0.23 p < 0.01
r = 0.05 p = 0.04
r = 0.08 p < 0.01
r = 0.82 p < 0.01
r = 0.71 p < 0.01
r = 0.62 p < 0.01
r = 0.60 p < 0.01
r = 0.41 p < 0.01
r = 0.43 p < 0.01
r = 0.27 p < 0.01
r = 0.25 p < 0.01
r = 0.67 p < 0.01
r = 0.73 p < 0.01
r = 0.70 p < 0.01
r = 0.32 p < 0.01
r = 0.38 p < 0.01
r = 0.20 p < 0.01
r = 0.22 p < 0.01
r = 0.76 p < 0.01
r = 0.80 p < 0.01
r = 0.62 p < 0.01
r = 0.63 p < 0.01
r = 0.46 p < 0.01
r = 0.41 p < 0.01
r = 0.84 p < 0.01
r = 0.58 p < 0.01
r = 0.60 p < 0.01
r = 0.42 p < 0.01
r = 0.41 p < 0.01
r = 0.55 p < 0.01
r = 0.62 p < 0.01
r = 0.48 p < 0.01
r = 0.45 p < 0.01
r = 0.93 p < 0.01
r = 0.78 p < 0.01
r = 0.73 p < 0.01
r = 0.81 p < 0.01
r = 0.76 p < 0.01
r = 0.88 p < 0.01

Figure S2 N.

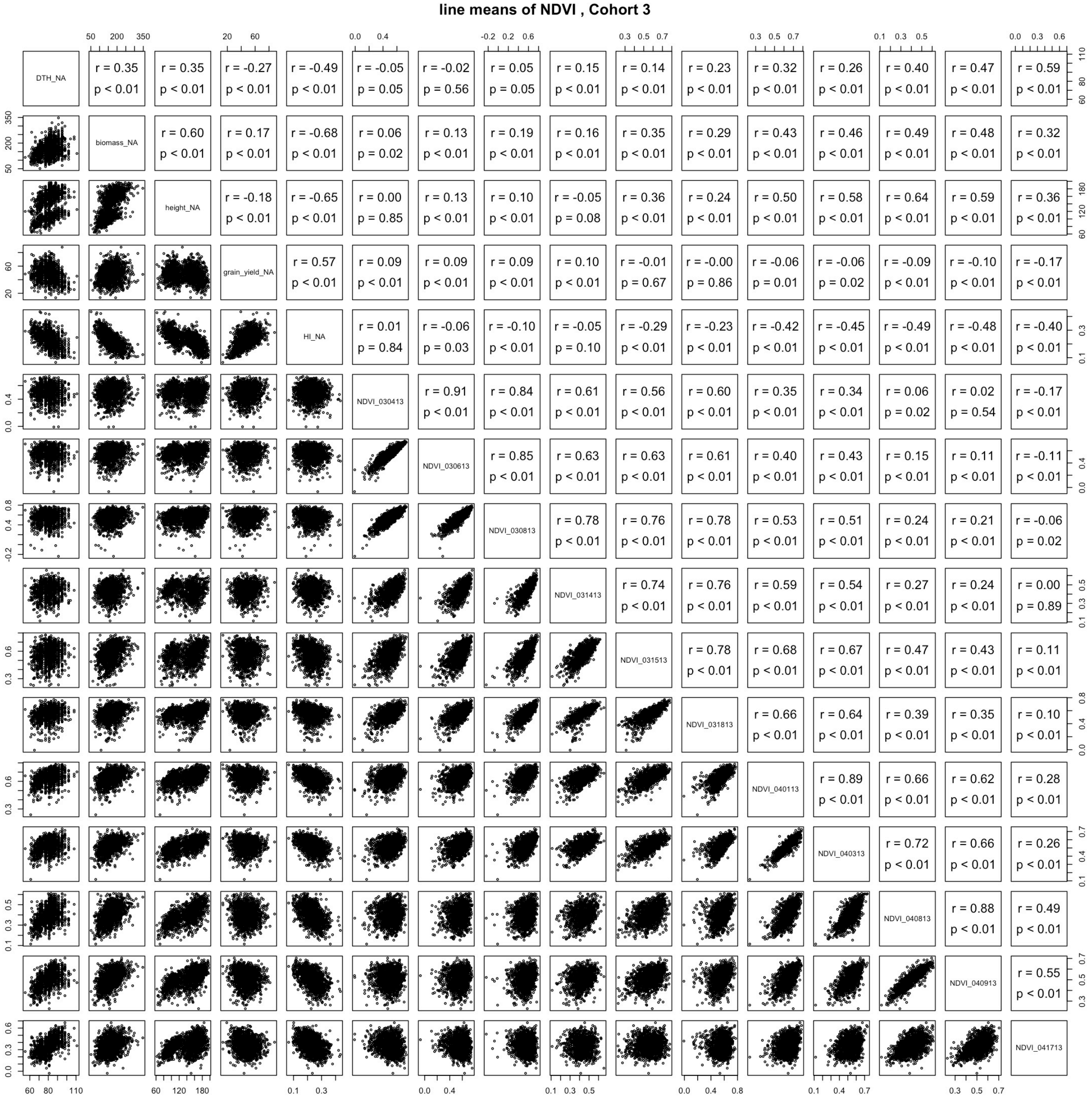

Supplementary Figure S2. Correlation matrix of each HTP sampling date plotted separately for each HTP trait and cohort. Trait and date (in MMDDYY format) are labeled in the diagonals. Correlations with manually measured traits (DTH, biomass & height for all three cohorts; grain yield & HI for Cohort 3 only) are included. In each panel, each HTP trait is presented separately for each cohort: A - Chla cohort 1, B - Chla cohort 2, C - Chla cohort 3, D - CTD cohort 1, E - CTD cohort 2, F - CTD cohort 3, G - HTPheight cohort 1, H - HTPheight cohort 2, I - HTPheight cohort 3, J - NDRE cohort 1, K - NDRE cohort 2, L - NDRE cohort 3, M - NDVI cohort 1, N - NDVI cohort 2, O - NDVI cohort 3.

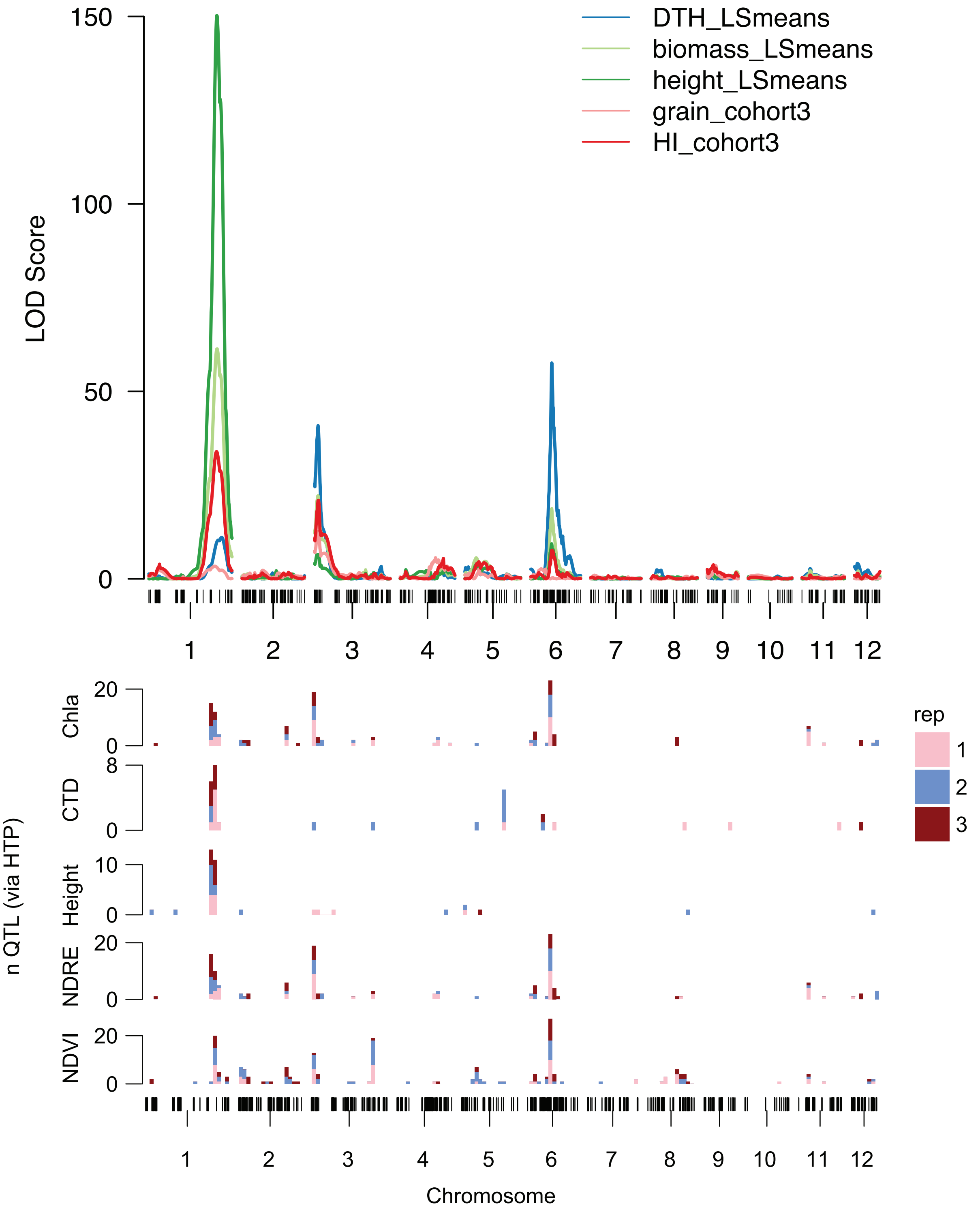

Supplementary Figure S3. Manually-measured traits are genetically correlated with HTP traits across QTL. Top panel plots QTL LOD scores for manually measured traits (LS means across three cohorts for days to heading, biomass, and height, and cohort 3 values for grain and harvest index). Bottom panel shows stacked histograms of the three cohorts for all statistically

significant QTL peak locations for five HTP traits across all 11 measurement days (N = 165 QTL models). See Table S4 for a list of all significant QTL.
